# Targeting TREX1 Induces Innate Immune Response in Drug-Resistant Small-Cell Lung Cancer

**DOI:** 10.1158/2767-9764.CRC-24-0360

**Published:** 2024-09-12

**Authors:** Takahiko Murayama, Navin R. Mahadevan, Catherine B. Meador, Elena V. Ivanova, Yuqiao Pan, Erik H. Knelson, Tetsuo Tani, Jun Nakayama, Xueying Ma, Tran C. Thai, Yin P. Hung, William Kim, Hideo Watanabe, Kathy Q. Cai, Aaron N. Hata, Cloud P. Paweletz, David A. Barbie, Israel Cañadas

**Affiliations:** 1 Nuclear Dynamics and Cancer Program, Fox Chase Cancer Center, Philadelphia, Pennsylvania.; 2 Cancer Epigenetics Institute, Fox Chase Cancer Center, Philadelphia, Pennsylvania.; 3 Department of Medical Oncology, Dana-Farber Cancer Institute, Boston, Massachusetts.; 4 Department of Pathology, Brigham and Women’s Hospital, Boston, Massachusetts.; 5 Division of Hematology/Oncology, Department of Medicine, Massachusetts General Hospital Cancer Center, Boston, Massachusetts.; 6 Belfer Center for Applied Cancer Science, Dana-Farber Cancer Institute, Boston, Massachusetts.; 7 Department of Oncogenesis and Growth Regulation, Osaka International Cancer Institute, Osaka, Japan.; 8 Laboratory of Integrative Oncology, National Cancer Center Research Institute, Tokyo, Japan.; 9 Department of Pathology, Massachusetts General Hospital Cancer Center, Boston, Massachusetts.; 10 Moores Cancer Center, UC San Diego, La Jolla, California.; 11 Center for Novel Therapeutics, UC San Diego, La Jolla, California.; 12 Department of Medicine, UC San Diego, La Jolla, California.; 13 Division of Pulmonary, Critical Care and Sleep Medicine, Department of Medicine, Icahn School of Medicine at Mount Sinai, New York, New York.; 14 Department of Genetics and Genomic Sciences, Icahn School of Medicine at Mount Sinai, New York, New York.; 15 Tisch Cancer Institute, Icahn School of Medicine at Mount Sinai, New York, New York.; 16 Histopathology Facility, Fox Chase Cancer Center, Philadelphia, Pennsylvania.

## Abstract

Small-cell lung cancer (SCLC) is the most lethal type of lung cancer. Paradoxically, this tumor displays an initial exquisite response to chemotherapy; however, at relapse, the tumor is highly resistant to subsequent available therapies. Here, we report that the expression of three prime repair exonuclease 1 (TREX1) is strongly induced in chemoresistant SCLCs. Assay for transposase-accessible chromatin using sequencing and chromatin immunoprecipitation sequencing revealed a significant increase in chromatin accessibility and transcriptional activity of TREX1 gene locus in chemoresistant SCLCs. Analyses of human SCLC tumors and patient-derived xenografts (PDX) also showed an increase in TREX1 expression in postchemotherapy samples. TREX1 depletion caused the activation of cyclic GMP-AMP synthase stimulator of interferon gene pathway due to cytoplasmic accumulation of damage-associated double-stranded DNA, inducing immunogenicity and enhancing the sensitivity of drug-resistant cells to chemotherapy. These findings suggest TREX1 upregulation may partially contribute to the survival of resistant cells, and its inhibition may represent a promising therapeutic strategy to enhance antitumor immunity and potentiate the efficacy of chemotherapy and/or immunotherapy in chemoresistant SCLCs.

**Significance:**

In this study, we show that targeting TREX1 induces an innate immune response and resensitizes SCLC cells to chemotherapy, representing a promising novel target for “immunologically” cold tumors, such as SCLC.

## Introduction

Small-cell lung cancer (SCLC) is a highly aggressive form of lung cancer characterized by high metastatic potential and poor clinical outcomes. SCLC initially shows a good response to first-line therapy with cisplatin/carboplatin and etoposide, but most patients relapse with chemoresistant tumors within 2 years of treatment (1–3). Although immunotherapy has shown promising advances in cancer treatment, SCLC continues to be a devastating disease, with only a small fraction of patients showing a response to these therapies (2, 4, 5). This is often because SCLC is characterized by reduced antigen presentation and an immunologically “cold” tumor microenvironment despite its high mutational burden (1, 6). Thus, there is an urgent need to identify novel therapeutic strategies to enhance antitumor immune responses in SCLC and other “cold” tumors, which display low immunogenicity and are poorly responsive to chemoimmunotherapy.

Accumulation of DNA in the cytosol can occur as a consequence of microbial infection, genotoxic stress, or genomic instability resulting in DNA damage (7). Cyclic GMP-AMP synthase (cGAS) stimulator of interferon gene (STING) functions as a sensing pathway that detects these cytosolic DNA species, triggering an antiviral inflammatory response that includes the activation of type I interferons and other genes involved in modulating the immune response. cGAS directly engages cytosolic DNA to trigger a signaling cascade that ultimately activates STING, which then coordinates the downstream transcriptional response via its effectors IRF3 and NF-κB (8, 9). However, mammalian cells have developed several strategies to prevent the accumulation of DNA in their cytosol or the activation of adaptive responses to these potentially threatening situations. One of these strategies involves the three prime repair exonuclease 1 (TREX1), the most abundant DNA 3′→5′ exonuclease in human cells (10), involved in the degradation of small DNA fragments to antagonize the cGAS-STING pathway and prevent severe autoimmunity. Importantly, mutations in TREX1 are often linked to autoimmune diseases, including Aicardi-Goutières syndrome and systemic lupus erythematosus (11, 12). However, although the role of TREX1 is well characterized in autoimmune diseases, its potential contribution to antitumor immunity and chemoresistance is poorly understood.

Recent studies have shown that chemotherapy can activate the cGAS-STING pathway in cancer cells, leading to an innate immune activation and recruitment of immune cells to the tumor microenvironment, which may contribute to the antitumor effects of these drugs (13, 14). Also, it has been reported that TREX1 expression is induced by high doses of radiation in different cancer cell lines to attenuate the immunogenicity derived from cytosolic DNA upon radiation (15). However, the specific role of the cGAS-STING pathway in chemoresistant cancer cells, as well as how cancer cells adapt and survive to the continuous accumulation of cytosolic DNA derived from chemotherapy treatment or chromosomal instability, remains to be elucidated.

In this study, we show that TREX1 expression is induced in human SCLC tumors and cell lines after DNA-damaging drug treatment. Depletion of TREX1 contributes to the derepression of tumor-intrinsic innate immune response by accelerating the accumulation of cytosolic dsDNA derived from DNA damage and leading to a cGAS-STING pathway activation. Importantly, targeting TREX1 sensitizes drug-resistant SCLC tumors to chemotherapy and T-cell recognition. Thus, our findings shed light on how SCLC, one of the deadliest tumors, resists chemotherapy, and we uncover a novel target to enhance the efficacy of chemotherapy and/or chemoimmunotherapy in SCLC and other “cold” tumors.

## Materials and Methods

### Cell lines and cell culture

The human SCLC cell lines NCI-H69, NCI-H69AR, and NCI-H82 were sourced from Dr. Joan Albanell’s laboratory and verified using short tandem repeat genotyping prior to experimental use. The RPP631 (RPP) SCLC mouse cell line and its mesenchymal, adherent subpopulation (RPP-A) were provided by Dr. Matthew Oser’s laboratory. We purchased cell lines NCI-H196, NCI-H1048, NCI-H2081, and HEK293T from the ATCC and used them in all experiments before they reached 10 passages. The NCI-H69M was derived from NCI-H69, as previously documented (16). Regular mycoplasma testing was performed on all cell lines, confirming the absence of contamination.

The SCLC cell lines H69, H69M, H69AR, H82, H1048, H2081, and H196 were cultured in RPMI-1640 containing 10% FBS (Hyclone), 2.5 g/L glucose, and 1× penicillin/streptomycin (P/S; 10,000 U/mL; Thermo Fisher Scientific). The HEK293T cells were maintained in DMEM containing 10% FBS and 1× P/S. The mouse cell lines, RPP and RPP-A were cultured in RPMI-1640 containing 10% FBS, 1× P/S, and HITES (1× insulin–transferrin–selenium, 10-nmol/L β-estradiol, and 10-nmol/L hydrocortisone). All cells were cultured in a humidified incubator containing 5% CO_2_ at 37°C.

### ATAC-seq and analysis

Assay for transposase-accessible chromatin using sequencing (ATAC-seq) experiment was performed on H69, H69M, and H69AR cells as detailed in a previous study (17). Briefly, 4.0 × 10^4^ to 5.0 × 10^4^ cells per replicate were collected, washed once with cold PBS, and lysed in 50 μL of cold lysis buffer (10-mmol/L Tris-HCl, 10-mmol/L NaCl, 3-mmol/L MgCl_2_, 0.1% IGEPAL CA630). The lysed nuclei were then incubated in a Tn5 transposition reaction mix. MinElute Reaction Cleanup kit (Qiagen; No. 28206) was used for purification. The ATAC-seq fragments were size-selected for those ranging from 115 to 600 bp using Pippin Prep 2% Agarose Gel Cassettes in conjunction with the Pippin Prep DNA Size Selection System (Sage Science). Following size selection, the ATAC libraries were amplified and ligated with Nextera sequencing primers using PCR. Finally, PCR primers were removed using the Agencourt AMPure XP bead (Beckman Coulter; No. A63880). Sequencing was performed on the Illumina HiSeq2000, after a library quality check with a Tapestation machine.

### H3K27Ac ChIP-seq analysis

Chromatin immunoprecipitation sequencing (ChIP-seq) was carried out as described previously (18). In brief, cells were cross-linked with 1% formaldehyde and subsequently washed in 5-mg/mL BSA in PBS. The cells were then resuspended in lysis buffer (50-mmol/L Tris-HCl, 10 mmol/L ethylenediaminetetraacetic acid (EDTA), 1% SDS, 1× protease inhibitor cocktail), and sonicated using the Covaris M220 sonicator to obtain fragments ranging from 100 to 1,000 bp. The fragment lengths were assessed using the Bioanalyzer DNA High Sensitivity Kit (Agilent). Fragmented chromatin was diluted in immunoprecipitation buffer (20-mmol/L Tris-HCl, 150-mmol/L NaCl, 2-mmol/L EDTA, 1% Triton X-100) and incubated with Dynabeads Protein G (Thermo Fisher Scientific; No. 10003D) precoated with anti-H3K27Ac (Abcam; No. ab4729) antibody. After washing the immunoprecipitates, DNA was treated with RNase A and Proteinase K on the beads and recovered in 1% SDS and 0.1-mol/L NaHCO_3_. The library was constructed with up to 10 ng collected DNA, with the NEBNext Ultra II DNA Library Prep Kit (New England Biolabs; No. E7645). Sequencing was conducted on the Illumina NextSeq500, capturing 38 nucleotides from each end of the paired reads.

### CRISPR-Cas9 gene editing vectors

Oligonucleotides coding for guide RNAs that target TREX1 and STING genes were designed using the single-guide RNA (sgRNA) designer (http://portals.broadinstitute.org/gpp/public/analysis-tools/sgrna-design). Sequences of sgRNA for human TREX1 #1 and #2 are 5′-TCT​GGA​TGG​TGC​CTT​CTGTG-3′ and 5′-GTC​CCC​TCC​AGA​CTC​GCA​CA-3′, respectively. #1 was used in most experiments. STING sgRNA sequence is 5′-GGT​ACC​GGG​GCA​GCT​ACT​GG-3′. For Scramble control (Scr), sgRNA from the Gecko library v2 was used as a dummy (5′-ATC​GTT​TCC​GCT​TAA​CGG​CG-3′). Lenti CRISPRv2 vectors were cloned as detailed in a previous study (19). Using X-treme Gene 9 DNA Transfection Reagent (Roche; No. XTG9-RO), lentiviral plasmids were introduced into HET293T cells alongside pMD2.G and psPAX2. 48 hours after transfection, the supernatant of transduced HEK293T cells was collected and filtered with a 0.45-μm filter.

### TREX1 expression vectors

The wild-type (WT) and catalytically deficient mutants (D18N, D200N) human TREX1 were cloned into the plx304 vector (control; plx304-NanoLuc). Lentiviral plasmids were transduced as previously described (20).

### Transduction of SCLC cells with lentiviral vectors

SCLC cell lines were infected with virus in the culture supernatant from transduced HEK293T cells, with 8-μg/mL polybrene (Santa Cruz Biotechnology; No. sc-134220). The cells were then centrifuged with the virus media at 2,000 rpm at 37°C for 2 hours, to enhance the efficiency of infection. After 24 hours, the virus media were replaced with complete RPMI-1640, and cells were cultured in a humidified incubator containing 5% CO_2_ at 37°C for an additional 24 hours. Following incubation, the virus-infected cells were selected with 1.0-μg/mL puromycin (Gibco; No. A11138-03) or 5.0-μg/mL blasticidin (Gibco; No. A11139-03). Lentiviral infection was performed whenever knockout cells were required, followed by collection of protein/RNA 7 days postselection.

### siRNA transfection

We purchased two different siRNA duplexes of human TREX1 (#1; s229446 and #2; s229447, with s229446 used in most experiments) and a nonspecific control siRNA duplex (siCtrl) with similar GC content (Silencer Select Negative Control No. 1 siRNA, no. 4390844) from Thermo Fisher Scientific. The siRNAs were transfected into cells, using RNAiMAX Transfection Reagent (Thermo Fisher Scientific; No. 13778500) in Opti-MEM I Reduced-Serum Medium (Gibco; No. 31985062),

### TREX1 immunofluorescent staining of clinical samples

Four-micron thick formalin-fixed, paraffin-embedded tissue sections were deparaffinized, incubated in 2% NaBH_4_ for the reduction of autofluorescence, and treated with the citrate-based (pH 6.0) Epitope Retrieval Solution. Slides were loaded into a Shandon Sequenza staining rack (Thermo Fisher Scientific; No. FIS73-310-017) and incubated in a 10% goat serum block overnight at 4°C. Alexa Fluor 647 Rabbit monoclonal (EPR14985) antibody to TREX1 (Abcam; No. ab209469) and FITC Mouse monoclonal (9C4) antibody to EpCAM (BioLegend; No. 324204) were diluted 1:100 and incubated for 1 hour at room temperature. VECTASHIELD Vibrance with 4,6-diamidino-2-phenylindole (Thermo Fisher Scientific; No. NC1601055) was used as the mounting medium.

Immunofluorescent images were acquired under uniform exposure settings using a 40× objective on a Nikon Eclipse 80i fluorescence microscope, which is equipped with an automated motorized stage (Proscan), a Z-stack system (Prior), and a Zyla 5.5 sCMOS camera (Andor). Image capture was carried out using the NIS-Elements AR software package.

Three tumor areas per sample were randomly selected by a board-certified pathologist based on EpCAM expression and nuclear morphology. TREX1 expression was then blindly scored on a 0 to 3+ scale using 0.5 increments.

### Immunoblotting

Protein lysates were prepared with RIPA buffer (Thermo Fisher Scientific; No. 89900) and the protein concentration was measured with the Pierce BCA Protein Assay Kit (Thermo Fisher Scientific; No. 23225). Protein extracts were subjected to polyacrylamide gel electrophoresis using the 4% to 12% NuPAGE gel system (Invitrogen; No. NP0322). Following electrophoresis, the proteins were then transferred to PVDF membranes, using iBlot2 Gel Transfer Device (Invitrogen). Transferred protein was immunoblotted using antibodies against TBK1 (no. 3013), S172 pTBK1 (no. 5483), IRF3 (no. 4302), S396 pIRF3 (no. 4947), STAT1 (no. 9172), Y701 pSTAT1 (no. 9171), STING (no. 13647), cGAS (no. 15102), β-actin (no. 3700; Cell Signaling Technology), human TREX1 (no. ab185228; Abcam), and mouse TREX1 (no. 611986; BD) after blocking with LICOR Blocking Buffer (LICOR; No. 927-60001).

IRDye 800CW Goat antimouse IgG (H+L; No. 926-32210) and IRDye 680RD Goat antirabbit IgG (H+L; No. 926-68071) were used as Secondary antibodies (LICOR Biosciences). To dilute primary and secondary antibodies, LICOR Antibody Diluent (no. 927-65001) was used. Phosho-specific antibodies were diluted in CanGet Signal Immunoreaction Enhancer Solutions (TOYOBO Co., Ltd. No. NKB-101) 1 (for primary) and 2 (for secondary). The blots were imaged using the LICOR Odyssey system.

### Immunocytochemistry

Cells were plated on BioCoat Culture Slide (Corning; No. 354630) and incubated overnight. The cells were fixed with 4% paraformaldehyde and permeabilized with 0.5% Triton X100 to facilitate the detection of protein expression in micronuclei. Cell samples were blocked using MAXblock Blocking Medium (Active Motif; No. 15252) and subsequently stained overnight with primary antibodies. Afterward, they were incubated for 1 hour with secondary antibodies, donkey antimouse IgG (H+L) conjugated with Alexa Fluor 488 (Invitrogen; No. A32766) and donkey antirabbit IgG (H+L) conjugated with Alexa Fluor 594 (Invitrogen; No. A32754). After washing, coverslips were mounted with ProLong Gold Antifade reagent with 4,6-diamidino-2-phenylindole (Invitrogen; No. P36935). Leica SP8 confocal microscope was used for obtaining immunofluorescence images. Antibodies against cGAS (No. 79978) were purchased from Cell Signaling Technology, and dsDNA antibody (no. ab27156) was purchased from Abcam.

### ELISA

According to the manufacturers’ instructions, IFNβ ELISA (R&D Systems; No. DIFNB0) and 2′,3′-Cyclic GAMP (cGAMP) ELISA (Arbor Assays; No. K067-H1) kits were used. Conditioned media from cells cultured for 72 hours with 5-µmol/L cisplatin or DMSO were collected and analyzed for secreted IFNβ. Cell lysates from cells cultured for 72 hours with 0.5-µmol/L reversine or DMSO were collected and analyzed for intra-cellular cGAMP.

Proteome Profiler Human Cytokine Array Kit (R&D Systems, no. ARY005B) was used for the assay of the cytokine array, according to the manufacturer’s instruction. Conditioned media was collected after 72 hours of treatment with 5-µmol/L cisplatin or DMSO.

### Flow cytometry analysis

Cells were collected, washed with PBS, and then stained with the antibodies anti-PDL1 (BioLegend; No. 329718), and anti-HLA-A, B, C (Biolegend; No. 311410). Corresponding isotype controls were used: no. 400232 for anti-PDL1 and no. 400220 for anti-HLA-A, B, C. The antibodies were diluted in PBS containing 2% FBS, to a final concentration of 2 μg/mL. The stained cells were evaluated on a BD LSR II Flow Cytometer, and the fluorescence intensity was compared with that of the isotype control antibodies. Flow cytometry data were analyzed using FlowJo software (TreeStar). Dead cells were identified and excluded using either PI staining or the Zombie NIR Fixable Viability Kit (BioLegend; No. 423106).

### RT-qPCR

Total RNAs were extracted from cells and purified using the RNeasy Mini Kit (Qiagen; No. 74106), following the manufacturer’s instructions. cDNA was generated from 1 μg of purified RNA using the SuperScript III First-Strand Synthesis SuperMix for quantitative PCR with reverse transcription (RT-qPCR) kit (Thermo Fisher Scientific; No. 18080-044). RT-qPCR for the indicated genes (gene names and primer sequences can be found in Supplementary Table S1) was performed using SYBR green PCR Master Mix (Applied Biosystems; No. 4367659) on the Applied Biosystems 7300 Fast real-time PCR system or the QuantStudio 6 Pro Real-Time PCR System and software. The expression data were analyzed with the –ΔΔCt relative quantification method. The expression of the housekeeping genes 36B4 (for human cells) or Actb (for mouse cells) was used to calculate the relative expression of genes of interest.

### RNA-seq

Total RNAs from NCI-H69AR cells, which were transfected with siCtrl or siTREX1 (#1), at day 3 posttransfection, were extracted and purified using the RNeasy Mini Kit (Qiagen; No. 74106). The quality of purified RNA was assessed using a 2100 Bioanalyzer RNA 6000 Nano assay (Agilent), and RNA concentration was determined using a Qubit 2.0 Fluorometer (Life Technologies). Illumina sequencing libraries were prepared using the NEBNext Ultra II Directional RNA Library Prep Kit for Illumina (NEB). The prepared libraries were sequenced on the Illumina NovaSeq 6000 by paired-end sequencing with a read length of 2 to 150 bp, with Novogene handling the sequencing process.

Using Kallisto, the expression levels for each gene were quantified from the sequencing data. The resultant data were summarized with the tximport package (ver. 1.18.0) of R software and RStudio (RStudio). Generated scaledTPM counts were used for further analysis as expression values for each gene. Gene set enrichment analysis (GSEA) was performed to identify gene signatures that are upregulated and downregulated in TREX1-depleted cells, compared with control cells.

### Proliferation assay

Cells were seeded 48 hours after siRNA transfection, in 12-well plates at low density (1,000–10,000 cells/well), and cultured in RPMI1640 with 10% FBS. Cells were harvested to enumerate the cell count after 2, 4, 6, and 8 days.

### Viability assay

Four thousand cells were plated onto 96-well plates and treated with cisplatin (10 nmol/L, 100 nmol/L, 1 µmol/L, 5 µmol/L, and 10 µmol/L) for 120 hours. Plates were read on the CLARIOstar Plus Microplate Reader (BMG Labtech) to obtain luminescent values of the CellTiter-Glo Cell Viability assay (Promega; No. G7571). DMSO-treated control cells were used to normalize. Analysis was performed using GraphPad Prism 7 Software. All conditions were evaluated in triplicate.

### Human SCLC xenograft

For the establishment of human SCLC cell xenografts, 1.0 × 10^6^ cells/mice (Scramble or sgTREX1) were subcutaneously implanted into the flank of NSG mice. Mice were maintained in pathogen-free facilities and housed in single-sex cages. When tumor volume reached 100 mm^3^, mice were challenged weekly with cycles of cisplatin (5 mg/kg IP, on day 1) as long as the mice did not lose >20% body weight when compared with the body weights at the point of the first treatment. One week was considered as one cycle. On the morning of day 1 of each week, mice were given 1.0 mL of normal saline subcutaneously to induce renal clearance of the cisplatin. Tumor size was measured and recorded every 2 to 3 days by digital caliper. Tumor volumes were calculated using the formula: volume = (length × width^2^)/2.

### Murine SCLC genetically engineered mouse model, cell line derivation, and tumor implantation studies

The RPP SCLC mouse cell line and its subpopulation RPP-A were established in the laboratory of Dr. Matthew G. Oser. The cells were initially sourced from SCLC tumors developed in LSL-Cas9 C57BL/6 mice that were intratracheally injected with AAV that encode Crerecombinase and sgRNAs targeting Trp53, Rb1, and Rbl2 (RPP) genes (21). Histopathologic examination of the tumors from which these cell lines were established confirmed the presence of SCLC.

To generate the syngeneic mouse tumor model, 8.0 × 10^6^ RPP-A cells, which were infected with Scramble control or sgTrex1-containing vector, were mixed with Matrigel (Corning; No. 354234) at a 1:1 ratio and were subcutaneously implanted into the flank of C57BL/6 mice. Tumor size was measured and recorded every 2 to 3 days using a digital caliper.

### Inducible knockdown of TREX1

To generate the inducible shTREX1 expressing cells, SMARTvector Tet-inducible TREX1 shRNA (horizon; No. V3IHSMCG) or control shRNA (horizon; No. VSC11652) were transduced into HET293T cells along with pMD2.G and psPAX2. H1048 cells were infected with the collected lentivirus. Doxycycline water, containing 5% sucrose and 2 mg/mL of doxycycline, was administered to all groups to induce knockdown of the TREX1 gene in H1048 cells, following the confirmation of palpable tumor formation. The doxycycline water was changed every 2 days.

### Generation of drug-resistant cells

Drug-resistant cells from H82 were raised by chronic exposure of the parental cells to gradually increasing cisplatin concentrations ranging from 0.25 to 1.0 µmol/L with each treatment lasting 72 hours, followed by a drug-free interval of 72 hours. Cells were maintained in the same concentration for at least four passages. The cell population that survived when the concentration reached 1.0 µmol/L was named H82-CispR cells.

### Cytoplasmic dsDNA quantification

Cytoplasmic fractions were isolated from nuclear fractions using the Nuclear Extract Kit (Active Motif; No. 40010) following the manufacturer’s instructions. Quantification of dsDNA in the cytoplasmic fraction was performed using the SpectraMax Quant AccuClear Nano dsDNA Assay kit (Molecular Devices; No. R8357). Plates were read on the CLARIOstar Plus Microplate Reader and software (BMG Labtech).

### TREX1 immunohistochemistry staining of PDX tumor samples

PDX tumors were established at Massachusetts General Hospital Cancer Center (22). The treatment history of PDX samples is summarized in Supplementary Table S2. mRNA expression data were downloaded from the Gene Expression Omnibus (GEO) database (GSE110853). Hematoxylin and eosin–stained sections were utilized for morphologic assessment, whereas 5-μm unstained sections were reserved for IHC studies.

IHC staining was performed using a VENTANA Discovery XT automated staining instrument (Ventana Medical Systems) with VENTANA reagents, following the manufacturer’s instructions. In brief, the slides were deparaffinized using EZ Prep solution (no. 950-102) for 16 minutes at 72°C. Epitope retrieval was achieved with CC1 solution (EDTA, pH 9.0.; No. 950-224) at high temperature for 32 minutes. Rabbit primary antibody: TREX1 was diluted at 1:50 (Abcam; No. 185228) using a TBS antibody diluent. Detection of the protein was carried out using the Ventana OmniMap antirabbit detection kit (no. 760-4311) and developed with the VENTANA ChromMap DAB detection kit (no. 760-159). The slides were then counterstained with hematoxylin II (no. 790-2208) for 8 minutes, followed by 4 minutes of treatment with Bluing reagent (no. 760-2037).

### TCR cloning, lentiviral infection, and T-cell coculture

TCR cloning, lentiviral infection, and T-cell coculture were performed, as previously reported (23). In brief, the Trbv29-Trbc1-P2A-Trav14D-3-DV8-Trac1 minigene was synthesized by Integrated DNA Technologies, using optimized constant regions. After cloning into the PLX_307 backbone vector, we transduced HEK293T cells using X-treme Gene 9 DNA Transfection Reagent. Forty-eight hours after transfection, the supernatant of transduced HEK293T cells was collected and filtered with a 0.45 μm filter. The virus media was applied to the target cells with 8-μg/mL polybrene. Cells were centrifuged with virus media at 2,000 rpm at 37°C for 2 hours to enhance the efficiency of infection. The infected cells were incubated at 37°C in a humidified incubator containing 5% CO_2_ for 24 hours, after which the virus media was replaced with complete RPMI-1640. 48 hours later, virus-infected cells were selected using 1.0-μg/mL puromycin. Following expansion, the puromycin-selected cells were sorted to achieve more than 98% purity using an antimouse pan-TCRβ antibody (Biolegend; No. 109211) expression.

Tumor cell–T-cell cocultures were performed at a stimulator/responder (S/R) ratio of 1:1. Tumor cells (1.0 × 10^5^) were seeded in a 24-well plate and stimulated with or without IFNγ (100 ng/mL) for 24 hours. Following stimulation, tumor cells were washed with PBS and subsequently replated in a fresh cell culture medium. After the wash, 1.0 × 10^5^ parental BW5147.3 or transgenic T cells were cocultured with tumor cells for 72 hours. After this incubation period, the cell-free supernatant was collected for ELISA analysis to measure IL2 levels.

### Statistical analyses

All graphs depict mean ± SEM. unless otherwise indicated. Differences between the two groups were assessed using either a two-tailed unpaired Student *t* test or a Mann–Whitney two-tailed test, as indicated in the figure legends. Where applicable, a two-way analysis of variance (ANOVA) was conducted, followed by Tukey’s multiple comparison test. Values of *, *P* < 0.01–0.05; **, *P* < 0.001–0.01; ***, *P* < 0.001–0.0001; or ****, *P* < 0.0001 were considered significant. GraphPad Prism 7 was used for statistical analysis of experiments, data processing, and presentation.

### Study approval

All human SCLC specimens were obtained from Dana–Farber Cancer Institute. This study was approved by the institutional review boards. Written informed consent was received from all participants before inclusion in the study.

All animal experiments were performed under protocols approved by the Institutional Animal Care and Use Committee at FCCC.

### Data availability

The H3K27Ac ChIP-seq data are available in the GEO repository (Accession Number: GSE168195). We also registered raw data from RNA sequencing (RNA-seq) experiments in the GEO repository (Accession Number: GSE272512). All other data will be available upon request from the corresponding author.

## Results

### TREX1 expression is induced in drug-resistant SCLC cells

**Figure 1:**
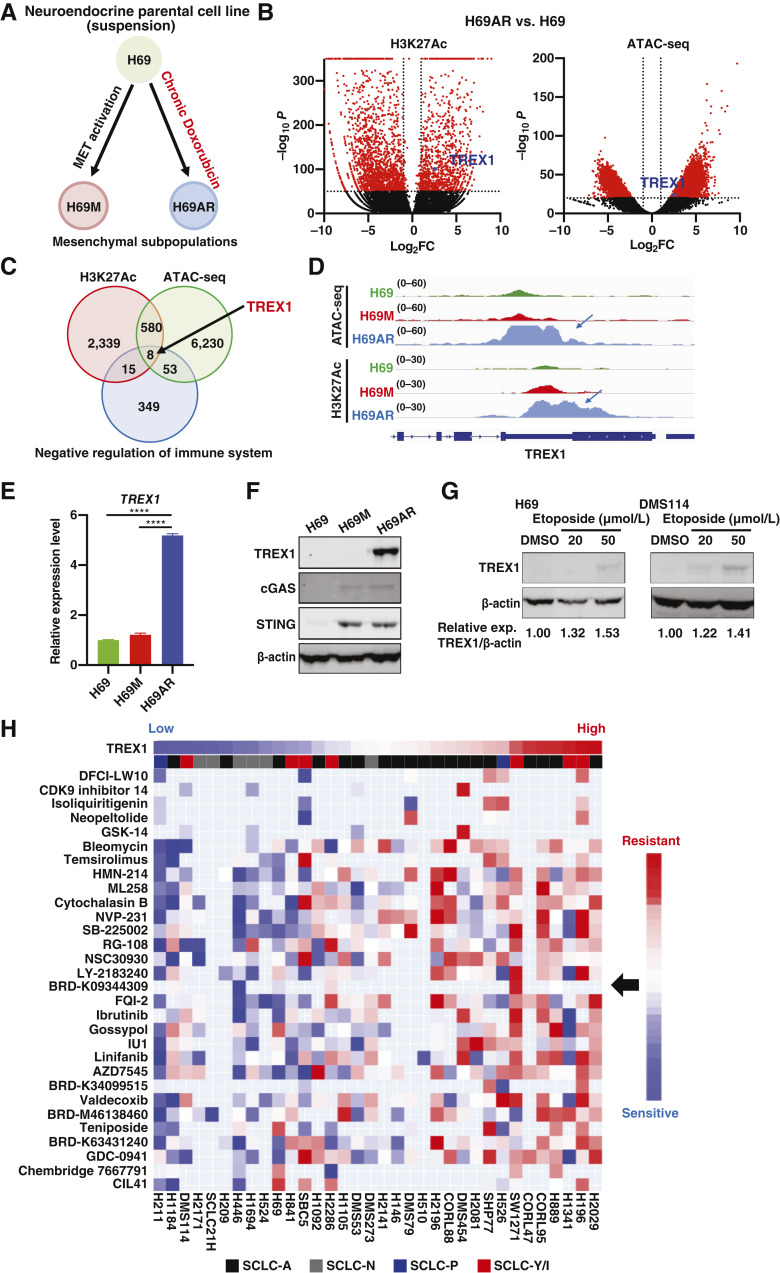
TREX1 expression is induced in drug-resistant SCLC cell lines. **A,** Schematic of establishment of subpopulations from H69 SCLC cell line. **B,** Volcano plots of H3K27Ac ChIP-seq and ATAC-seq analyses comparing H69AR and H69 cells are shown. **C,** Venn diagrams for top-hits genes of H3K27Ac ChIP-seq and ATAC-seq, and genes listed in “GOBP_NEGATIVE_REGULATION_OF_IMMUNE_SYSTEM_PROCESS.” TREX1 was selected as one of the eight overlapped genes. **D,** ATAC-seq and H3K27Ac ChIP-seq analysis to compare H69, H69M, and H69AR cells. Results of the *TREX1* gene region are shown. **E,** Expression levels of the *TREX1* gene in H69, H69M, and H69AR cells were compared by qPCR (mean ± SEM; *n* = 3). **F,** Expression levels of TREX1, cGAS, and STING in H69, H69M, and H69AR cells were compared by immunoblotting. **G,** Expression levels of TREX1 in H69, DMS114 cells treated with etoposide (20 or 50 µmol/L) or DMSO were compared by immunoblotting. **H,** Drug sensitivity/resistance data of SCLC cell lines are summarized. Cells are lined up based on *TREX1* expression levels. Data represent mean ± SEM. ns, not significant; *, *P* < 0.05; **, *P* < 0.01; ***, *P* < 0.001; ****, *P* < 0.0001 by one-way ANOVA followed by Dunnett’s multiple comparisons test (**E**).

To systematically investigate which epigenetic changes could contribute to the chemoresistance of SCLCs, and to the suppression of innate immunity in the resistant cells, we performed ATAC-seq and H3K27 acetylation (Ac) ChIP-seq in the established human cell line NCI-H69 and two isogenic nonneuroendocrine derivatives, H69AR and H69M subpopulations (Fig. 1A). H69AR is a multidrug-resistant cell line established by culturing H69 cells with gradually increasing doses of adriamycin for 14 months (24, 25), whereas H69M cells were previously established by our group by continuously exposing H69 cells to hepatocyte growth factor (16, 26). Among the top genes enriched for chromatin accessibility and H3K27Ac gain in H69AR chemoresistant cells compared with H69 cells (Fig. 1B), we extracted genes that are also known to negatively regulate immune response (Fig. 1C) and identified the three prime repair exonuclease 1 (TREX1) as a potential negative regulator of immune response in chemoresistant cells. Interestingly, chromatin accessibility and H3K27Ac gain were dramatically increased in H69AR chemoresistant cells compared with H69 and H69M cells (Fig. 1D). We also confirmed a significant increase in TREX1 mRNA and protein levels in H69AR cells when compared with H69 and H69M, as well as cGAS and STING protein expression. Interestingly, H69M cells, which were not exposed to chemotherapeutic agents, lacked TREX1 expression but also expressed cGAS and STING (Fig. 1E and F). This might be because mesenchymal and EMT markers, which are expressed in H69M cells, are tightly correlated with STING expression (27). Because TREX1 is known to play a crucial role in regulating the innate immune response, these data suggest that TREX1 induction may be a mechanism for SCLC cells to adapt and survive the inflammation associated with DNA-damaging drug treatments such as chemotherapy.

Next, we evaluated whether chemotherapy treatment induced in drug-resistant SCLC cell lines directly contributes to TREX1 upregulation in SCLC cells. We treated H69 cells with etoposide, which has been widely used for SCLC treatment in combination with cisplatin/carboplatin, and tested TREX1 protein levels. As we expected, TREX1 expression was induced upon etoposide treatment in a dose-dependent manner, as well as *IFNB* gene upregulation (Fig. 1G; Supplementary Fig. S1A). Because H69 cells were established from a patient with SCLC previously treated with chemotherapy, we also tested this in DMS114 cells, a cell line derived from an untreated patient with SCLC, and confirmed that etoposide treatment also increased TREX1 expression in DMS114 cells (Fig. 1G; Supplementary Fig. S1A). We then transfected H69AR and NCI-H1048 cells with poly (dA:dT) or poly (I:C), analogs of dsDNA and double-stranded RNA (dsRNA), respectively, and tested TREX1 expression (Supplementary Fig. S1B). dsDNA and dsRNA analogs caused upregulation of TREX1, suggesting that activation of the innate immune response can induce TREX1 expression in SCLC cells, which is consistent with previous reports (15, 28).

To investigate the association between TREX1 and chemoresistance in SCLC further, we next evaluated TREX1 mRNA expression levels across human SCLC cell lines with available drug-response data from the Cancer Cell Line Encyclopedia. Importantly, we found that TREX1 high-expressing SCLC cell lines tend to show multidrug resistance when compared with TREX1 low-expressing cells (Fig. 1H; Supplementary Fig. S1C). Given that SCLC has been recently classified in four different transcriptional subtypes defined by ASCL1, NEUROD1, POU2F3 (SCLC-A, SCLC-N, and SCLC-P) or YAP1 (SCLC-Y; or characterized by low expression of ASCL1, NEUROD1, and POU2F3 and accompanied by an Inflammatory gene signature; SCLC-I; refs. 1, 29), we next evaluated the expression of TREX1 across these different SCLC molecular subtypes. Although SCLC-N tended to show lower expression of TREX1, we did not observe significant differences between neuroendocrine (NE; SCLC-A and -N) and non-NE (SCLC-P and -Y/I) subtypes (Supplementary Fig. S1C and S1D). However, we also evaluated TREX1 expression in SCLC cell lines grouped as “pre” (cell lines derived from patient prior to chemo) or “post” (cell lines derived from patients previously treated) and observed that TREX1 expression tended to be higher in “post” cell lines suggesting that, beyond transcriptional subtypes, TREX1 upregulation might be an adaptive strategy for SCLC cells to adapt and survive to the continuous accumulation of cytosolic DNA derived from DNA-damaging agents (Supplementary Fig. S1E). Collectively, these data confirmed that TREX1 is induced after chemotherapy treatment and associated with chemoresistance in SCLC.

### TREX1 depletion induces a tumor-intrinsic innate immune response and enhances immunogenicity in drug-resistant SCLC cells

**Figure 2:**
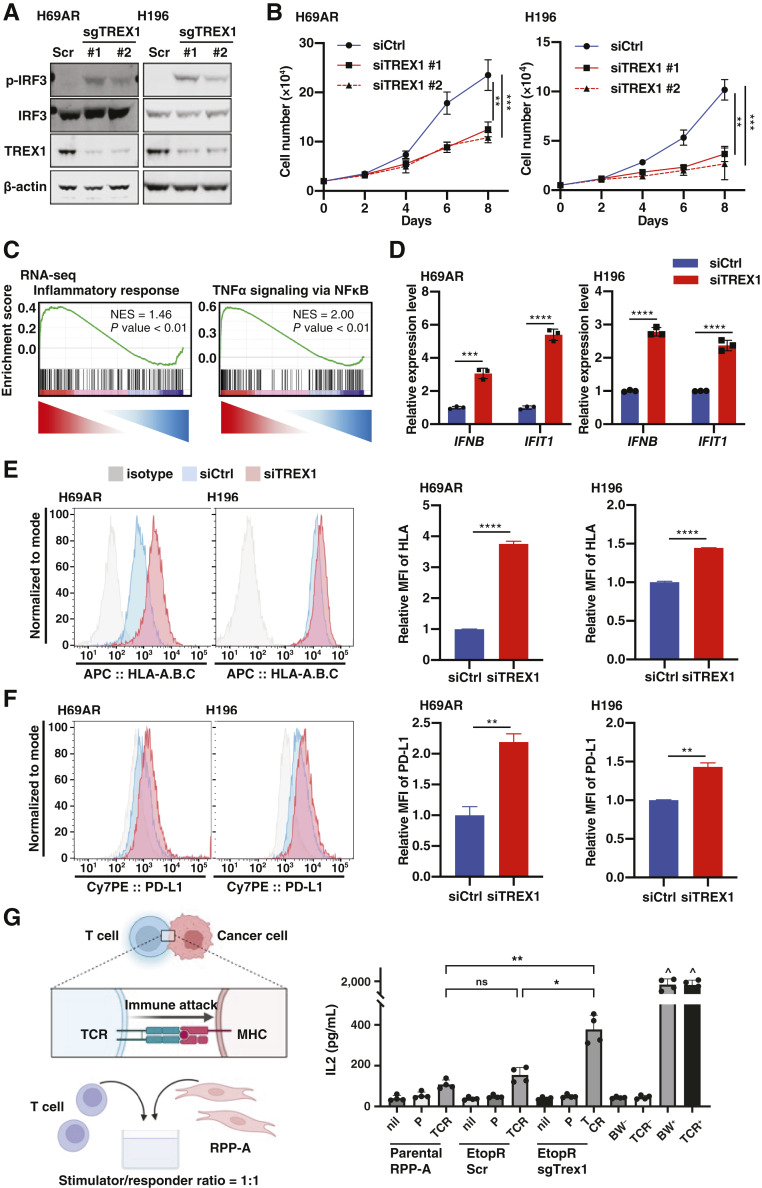
TREX1 depletion suppresses SCLC growth and induces IFN response. **A,** Expression levels of TREX1, IRF3, and p-IRF3 in H69AR and H196 were compared by immunoblotting between cells transduced with Scr and sgTREX1 (#1 and #2). **B,** Growth curves of H69AR and H196 cells were compared between siCtrl and siTREX1 (#1 and #2; mean ± SEM; *n* = 3). **C,** GSEA analysis with H (hallmark) gene sets, based on RNA-seq results of siTREX1 vs. siCtrl H69AR cells. **D,** Expression levels of *IFNB* and *IFIT1* genes in cells transfected with siCtrl and siTREX1 (#1) were compared by qPCR (mean ± SEM; *n* = 3). **E** and **F,** HLA-A, HLA-B, and HLA-C (**E**) and PDL1 (**F**) expressions in H69AR and H196 cells transfected with siCtrl or siTREX1 were compared by flow cytometry analysis. Data are representative of three independent experiments (Left). Mean fluorescence intensity was quantified by FlowJo (Right; *n* = 3). Cells were analyzed 3 days after siRNA transfection. **G,** (Left) Schematic of cancer cell–T-cell coculture, performed at a stimulator/responder (S/R) ratio of 1:1. (Right) IL2 secretion from 72 hours cocultures of RPP-A-EtopR (Scr or sgTrex1) or parental RPP-A cells ± 24-hour IFNγ (100 ng/mL) stimulation and no (nil), parental untransduced BW5147.3 (P), or Trav14D-3-DV8; Trbv29 transgenic BW5147.3 (TCR) T cells. BW (−/+) and TCR (−/+) bars represent P or TCR T cells alone without (−) or with (+) PMA (50 nmol/L) and ionomycin (1 μg/mL). ^, above assay threshold of detection (mean ± SEM; *n* = 4). Data represent mean ± SEM. ns, not significant; *, *P* < 0.05; **, *P* < 0.01; ***, *P* < 0.001; ****, *P* < 0.0001 by unpaired Student *t* test (**D**, **E** and **F**), one-way ANOVA followed by Dunnett’s multiple comparisons test (**B**) and two-way ANOVA followed by Tukey’s multiple comparisons test (**G**).

Based on previous studies reporting that TREX1 degrades cytoplasmic DNA and inhibits autoimmune diseases (30, 31), we hypothesized that adaptive resistance to chemotherapy (and possibly chemoimmunotherapy) could involve TREX1 induction as a means of suppressing innate immune activation due to accumulation of dsDNA or other forms of chemotherapy-induced DNA damage. To investigate the effects of TREX1 depletion in chemoresistant SCLC cell lines, we utilized CRISPR-Cas9 system-mediated TREX1 knockout (sgTREX1) in H69AR and NCI-H196 cells (Fig. 2A), both of which showed higher drug resistance than H69 cells (Supplementary Fig. S2A). Indeed, H69 cells exhibited a higher ratio of cytoplasmic DNA increase after etoposide treatment compared with H69AR or H196, both of which express elevated levels of TREX1 under basal conditions (Supplementary Fig. S2B). We observed that TREX1 depletion induced an increase of p-IRF3 (Fig. 2A), which was also confirmed by using two different siRNAs to target TREX1 mRNA (siTREX1; Supplementary Fig. S2C and S2D). We also found a significant decrease in cell proliferation in both cell lines after TREX1 depletion (Fig. 2B). Similar effects on cell growth were also observed in NCI-H2081 (SCLC-A subtype) and H1048 (SCLC-P subtype) cells (Supplementary Fig. S2E and S2F), whereas no significant effect was observed on HEK293T and FC1010 cells (Supplementary Fig. S2G and S2H), suggesting that TREX1 plays a crucial role in the survival and growth of drug-resistant cells.

To evaluate whether TREX1 upregulation is involved in the suppression of an innate immune response in chemoresistant SCLC, we performed RNA-seq on siCtrl and siTREX1 H69AR cells. As we expected, GSEA revealed that the hall mark gene sets of inflammatory response and TNFα signaling were strongly upregulated in TREX1-depleted cells (Fig. 2C; Supplementary Fig. S2I). To validate this, we tested the expression of IFN and IFN-stimulated genes (ISG) in SCLC cells after TREX1 depletion by RT-qPCR. We detected that loss of TREX1 significantly induced the expression of *IFNB* and *IFIT1* in multiple cell lines tested (Fig. 2D; Supplementary Fig. S2J). Furthermore, we observed an enrichment of a STING signaling gene set in TREX1-depleted cells (Supplementary Fig. S2K), suggesting that the IFN response may be derived from cGAS-STING pathway activation. Given that SCLC is characterized by a low or absent expression of MHC class I (MHC-I; ref. 32), we next examined the impact of TREX1 depletion on cell-surface expression of MHC class I proteins by flow cytometry. Interestingly, loss of TREX1 induced a significant increase of MHC class I proteins (HLA-A, HLA-B, and HLA-C) after TREX1 loss in H69AR and H196 cells (Fig. 2E). Also, PDL1 expression was upregulated after TREX1 deletion in both cell lines (Fig. 2F). Together, these data indicate that TREX1 loss activates a tumor intrinsic innate immune response, probably caused by an aberrant accumulation of chemotherapy-derived cytoplasmic DNAs, which lead to the decreased growth rate of drug-resistant cells.

The induction of an innate immune response and the increase in antigen presentation by TREX1 loss (Fig. 2C–F) suggests that an increased immunogenicity may influence the tumor microenvironment and provoke antitumor immune responses. To test this hypothesis, we took advantage of the mouse SCLC cell lines established from a CRISPR-derived Tp53, Rb1, Rbl2 (RPP) SCLC genetically engineered mouse model (21, 23). We used RPP cells, classical neuroendocrine SCLC cells, and isogenic nonneuroendocrine adherent derivative, RPP-A cells. Interestingly, RPP-A cells exhibited TREX1 overexpression when compared with RPP cells (Supplementary Fig. S2L). We tried to generate chemoresistant mouse SCLC cells treating RPP and RPP-A cells with cisplatin and etoposide. Although we were unable to generate chemoresistant RPP cells, we successfully established an etoposide-resistant RPP-A clone (RPP-A-EtopR). To do so, we treated RPP-A cells with a high dose of etoposide (IC90) for 24 hours, and then, we kept them in culture for 1 month (Supplementary Fig. S2M). As we expected, we confirmed increased TREX1 levels in RPP-A-EtopR cells compared with parental RPP-A cells (Supplementary Fig. S2N). Furthermore, CRISPR-mediated deletion of TREX1 in RPP-A-EtopR cells induced the expression of ISGs in a STING-dependent manner (Supplementary Fig. S2O), suggesting a tumor-intrinsic innate immune response.

Next, to directly interrogate the impact of TREX1 depletion on the intrinsic immunogenicity of chemoresistant RPP-A cells, as well as the influence on the tumor microenvironment, we performed subcutaneous inoculation of Scr and sgTrex1 RPP-A-EtopR cells into syngeneic C57BL/6 mice. However, Scr and sgTrex1 tumors were spontaneously rejected and we were unable to use this model to investigate the impact of TREX1 deletion on tumor growth and immune infiltration (Supplementary Fig. S2P). These results are consistent with a previous report also showing that RPP-A cells are rapidly spontaneously rejected *in vivo* in an adaptive immune-dependent manner soon after inoculation and heavily infiltrated with immune cells, particularly CD8 effector T cells (23). Therefore, we then assessed whether the tumor-intrinsic immune signaling, including increased MHC-I antigen presentation, induced in RPP-A-EtopR cells following TREX1 deletion can lead to T cell activation *in vitro*. We took advantage of an immunodominant TCR clone previously identified within the CD8 T cell population of RPP-A tumors (Trav14D-3-DV8, Trbv29; ref. 23) and we cloned the Trav14D-3-DV8, Trbv29 CD8 TCR into the TCR-null line BW5147.3. We then cocultured Trav14D-3-DV8, Trbv29-expressing T cells with RPP-A-EtopR cells (Scr vs. sgTrex1) for 72 hours to evaluate *in vitro* T-cell recognition (Fig. 2G). Interestingly, TREX1-depleted RPP-A-EtopR cells induced a significantly higher activation of Trav14D-3-DV8, Trbv29-expressing T cells when compared with Scr cells. Taken together, these data suggest that targeting TREX1 in chemoresistant SCLC cells has the potential to increase tumor cell immunogenicity capable of evoking CD8 T-cell activation.

### TREX1 depletion leads to cGAS-STING pathway activation in drug-resistant SCLC cells

**Figure 3:**
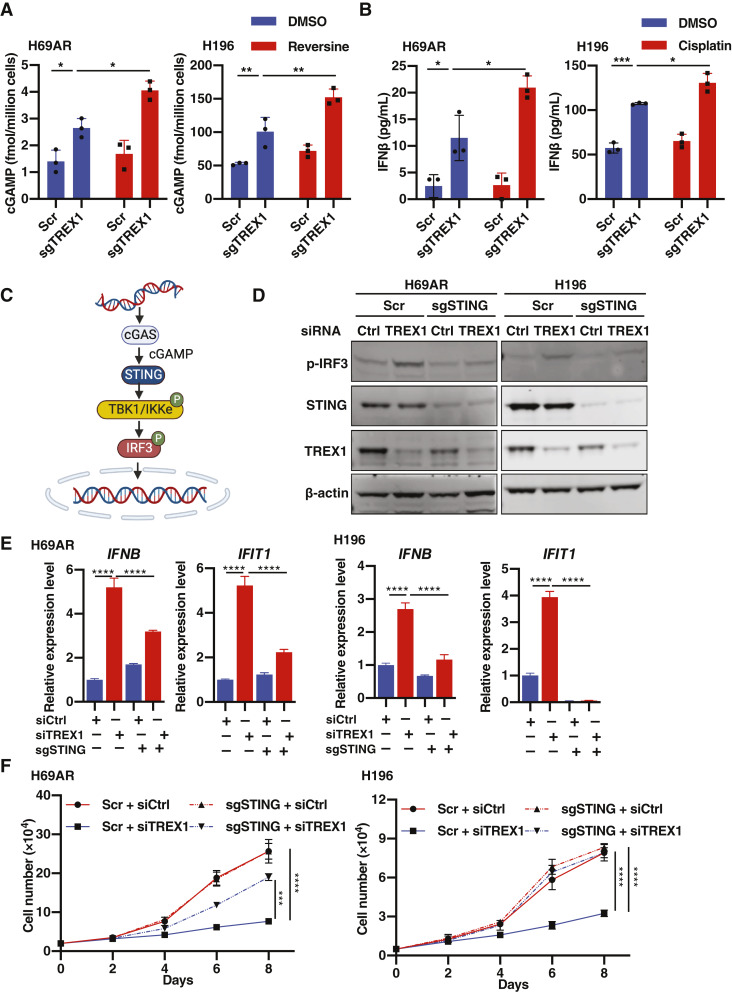
Immunogenicity is induced in TREX1 depleted cells through cGAS-STING activation. **A,** ELISA of human cGAMP level in cell lysates of Scr or sgTREX1 H69AR and H196 cells, treated with 0.5 µmol/L reversine or DMSO for 72 hours. **B,** ELISA of human IFNβ level in conditioned medium derived from Scr or sgTREX1 H69AR and H196 cells, treated with 5-µmol/L cisplatin or DMSO for 72 hours. **C,** Schematic of cGAS-STING pathway leading to IFN response. STING is a crucial mediator of the dsDNA sensing pathway. **D,** Expression levels of TREX1, STING, and p-IRF3 in H69AR and H196 cells were compared by immunoblotting between cells transfected with siCtrl and siTREX1 (#1) in Scr or sgSTING background. **E,** Expression levels of *IFNB* and *IFIT1* genes were compared between cells transduced with Scr or STING sgRNA, after transfection with siCtrl and siTREX1 (#1; mean ± SEM; *n* = 3). **F,** Growth curves of H69AR and H196 cells [Scr or sgSTING, transfected with siCtrl or siTREX1 (#1)] were compared (mean ± SEM; *n* = 3). Data represent mean ± SEM. ns, not significant; *, *P* < 0.05; **, *P* < 0.01; ***, *P* < 0.001; ****, *P* < 0.0001 by two-way ANOVA followed by Tukey’s multiple comparisons test (**A**, **B**, **E**, and **F**).

We next investigated how TREX1 depletion induces an innate immune response in chemoresistant SCLC cells. Given that TREX1 degrades dsDNA in the cytoplasm, particularly in micronuclei in which cGAS senses aberrant dsDNA accumulation (33), we then evaluated whether there were any variations in the accumulation of dsDNA and the recruitment of cGAS in micronuclei in SCLC cells after CRISPR-mediated TREX1 knockout. The deletion of TREX1 did not result in any significant change in the number of micronuclei or the accumulation of cGAS (Supplementary Fig. S3A), suggesting that TREX1 is not suppressing micronuclei formation or preventing the recruitment of cGAS to micronuclei. This observation is consistent with a previous report (34) showing that cGAS and TREX1 recruitment to micronuclei is dependent on the integrity of micronuclear envelope, and TREX1 can inhibit cGAS activation, but not recruitment, by degrading dsDNA in micronuclear envelope–ruptured micronuclei. Thus, to evaluate cGAS activity after TREX1 loss in our model, we measured cGAMP levels by ELISA in SCLC cells. This analysis showed that sgTREX1 cells accumulated a significantly higher amount of cGAMP when compared with Scramble (Scr) control cells (Fig. 3A), indicating that TREX1 plays a critical role in suppressing cGAMP production by cGAS in chemoresistant SCLC cells. This result is consistent with a significant increase of dsDNA accumulation in the cytoplasm observed in TREX1-depleted cells (Supplementary Fig. S3B). We also observed a further increase of cGAMP production when TREX1-depleted cells were treated with the MPS1 inhibitor reversine (Fig. 3A), which is known to cause DNA damage by inhibiting the spindle assembly checkpoint and inducing replication stress (34). Furthermore, ISG genes were further induced by reversine treatment in TREX1-depleted cells, indicating that cGAMP accumulation leads to STING activation (Supplementary Fig. S3C). These data further support the idea that TREX1 degrades aberrant cytoplasmic DNA to suppress the cGAS-STING pathway in chemoresistant SCLC cells.

To further investigate TREX1-mediated regulation of tumor intrinsic innate immune responses in chemoresistant SCLC, we next asked whether a type I IFN response was induced after TREX1 depletion by measuring secretion of IFNβ protein in the conditioned media of sgTREX1 and Scr cells. ELISA analysis showed that IFNβ accumulation was significantly higher in TREX1-depleted cells (Fig. 3B), which was further increased by treatment with the DNA-damaging agent cisplatin, indicating that the accumulation of cytoplasmic dsDNA caused by damaged genomic DNA contributes to the immune response occurred after TREX1 loss. To investigate this further, we evaluated the secretion of multiple cytokines and chemokines by a Luminex cytokine/chemokine array assay in sgTREX1 and Scr-conditioned medium. Indeed, TREX1 loss strongly induced the secretion of multiple cytokines and chemokines in H69AR and H196 cells (Supplementary Fig. S3D). In line with the IFNβ results above, cytokine/chemokine secretion was further induced by cisplatin treatment in TREX1-depleted cells (Supplementary Fig. S3D). Together, these results suggest that targeting TREX1 is a promising strategy to induce a tumor-intrinsic innate immune response in drug-resistant SCLC by derepressing dsDNA-cGAS-STING pathway activation.

To assess whether aberrantly accumulated cytoplasmic DNA is the main contributor to the innate immune activation observed in TREX1-depleted cells, we inhibited dsDNA-cGAS-STING pathway activation in SCLC cells through CRISPR-mediated deletion of STING (Fig. 3C). As expected, TREX1 loss induced phosphorylation of IRF3, as well as upregulation of *IFNB* and *IFIT1* in Scr cells. In contrast, p-IRF3, *IFNB*, and *IFIT1* induction was abrogated in sgSTING cells after TREX1 loss (Fig. 3D and E; Supplementary Fig. S3E). Interestingly, STING depletion also led to a near-complete restoration of cell proliferation rates after TREX1 loss (Fig. 3F), further suggesting that the innate immune response triggered by dsDNA-cGAS-STING pathway activation is contributing to the cell growth suppression observed in TREX1 depleted cells. Furthermore, HLAs and PDL1 expression levels were also strongly decreased in STING-depleted cells after TREX1 loss when compared with control cells (Supplementary Fig. S3F and S3G). Collectively, these data suggest that TREX1 depletion in chemoresistant SCLC cells contributes to the induction of a tumor intrinsic innate immune response by activating the cGAS-STING pathway, which senses chemotherapy-derived dsDNA aberrantly accumulated in the cytoplasm.

### TREX1 depletion increases the sensitivity of drug-resistant SCLC cells to chemotherapy

**Figure 4:**
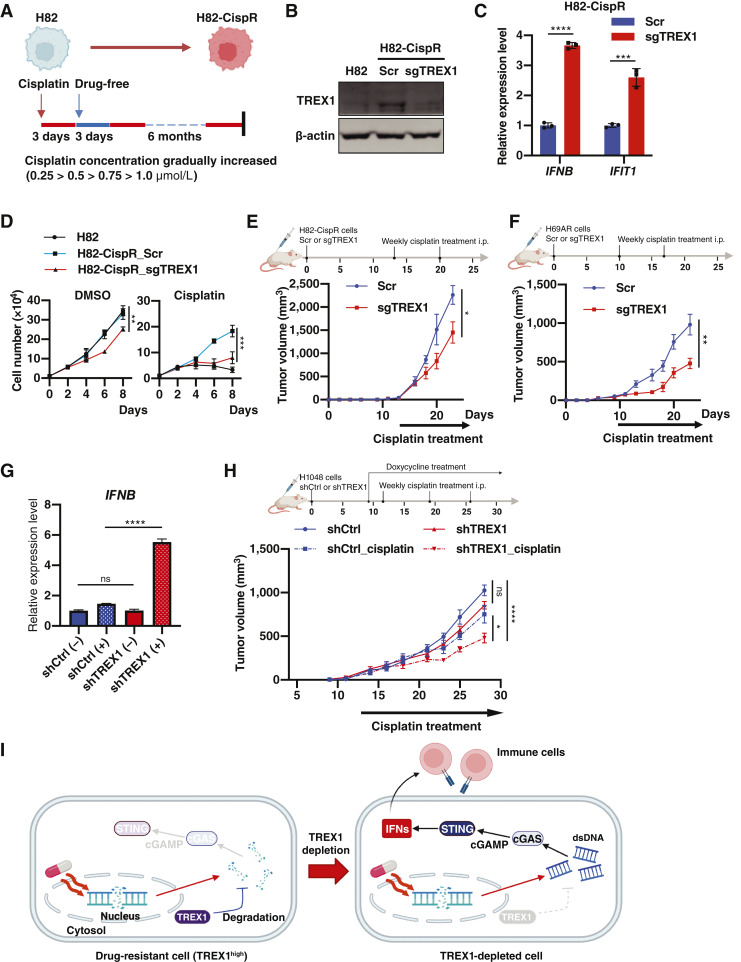
TREX1 depletion resensitizes resistant SCLC tumors to chemotherapy. **A,** Schematic of the method used to establish cisplatin-resistant H82 (H82-CispR) cells. **B,** Expression levels of TREX1 in H82 and H82-CispR (Scr and sgTREX1) cells were compared by immunoblotting. **C,** Expression levels of *IFNB* and *IFIT1* genes were compared between Scr and sgTREX1 H82-CispR cells by qPCR (mean ± SEM; *n* = 3). **D,** Growth curves of the indicated cells treated with DMSO (Left) or 1.0-µmol/L cisplatin (Right) were compared (mean ± SEM; *n* = 3). **E,** Tumor growth curves of Scr and sgTREX1 H82-CispR tumors (*n* = 5), treated with 5-mg/kg cisplatin, were compared. **F,** Tumor growth curves of Scr and sgTREX1 H69AR tumors (*n* = 6), treated with 5-mg/kg cisplatin, were compared. **G,** Expression levels of the *IFNB* gene were compared between shCtrl and shTREX1 H1048 cells treated w/wo DOX (mean ± SEM; *n* = 3). **H,** Tumor growth curves of shCtrl and shTREX1 H1048 tumors (*n* = 6), treated with 5-mg/kg cisplatin, were compared. **I,** Schematic model of antitumor effects caused by TREX1 depletion. Data represent mean ± SEM. ns, not significant; *, *P* < 0.05; **, *P* < 0.01; ***, *P* < 0.001; ****, *P* < 0.0001 by unpaired Student *t* test (**C**, **E**, and **F**) and one-way ANOVA followed by Dunnett’s multiple comparison test (**D**), and two-way ANOVA followed by Tukey’s multiple comparison test (**G** and **H**). (**I,** Created with BioRender.com.)

To better understand whether TREX1 upregulation is a common mechanism in SCLC cells to suppress cGAS-STING pathway activation due to accumulation of chemotherapy-induced dsDNAs or other forms of DNA damage, we established a new cisplatin-resistant human SCLC cell line *in vitro*. We used the well-established human SCLC cell line NCI-H82, which showed very low levels of TREX1 protein expression. We treated H82 cells with gradually increasing doses of cisplatin (Fig. 4A) for 6 months and we confirmed that the established resistant cells (H82-CispR) showed higher resistance to cisplatin compared with parental H82 cells (Supplementary Fig. S4A). Importantly, H82-CispR cells showed a strong induction of TREX1 expression, at mRNA and protein levels, compared with parental H82 cells (Fig. 4B; Supplementary Fig. S4B). Consistent with results in H69AR and H196 cell lines (Fig. 2D), TREX1 depletion also induced expression of ISGs (Fig. 4B and C).

To assess whether TREX1 upregulation contributes to cisplatin resistance in H82-CispR cells, we tested cell viability in H82-CispR cells after TREX1 depletion (Fig. 4D). As we expected, TREX1 loss increased the sensitivity of H82-CispR cells to cisplatin at a similar level as parental H82 cells, whereas H82-CispR Scr cells were resistant [Fig. 4D (Right)]. Interestingly, TREX1 depletion also decreased cell viability in H82-CispR in the absence of cisplatin treatment [Fig. 4D (Left)], suggesting that TREX1 also plays a crucial role in the survival and growth of cisplatin-resistant cells. To further explore whether TREX1 overexpression could induce chemoresistance by itself or additional mechanisms are involved, we next stably transduced DMS114 cells (originally derived from a patient with chemonaïve SCLC) and H82 (derived from a patient previously treated with chemotherapy) with TREX1-WT or TREX1 mutant constructs and evaluated cisplatin and etoposide effects on cell viability. Consistent with a previous report (35), exogenous TREX1 expression was not sufficient to confer chemoresistance in these cells and we did not observe significant changes in cell viability when compared with empty vector control (Supplementary Fig. S4C). This indicates that some other factors, including drug efflux pumps, may be necessary to confer chemoresistance, as we observed *ABCC3* and *ABCC6* upregulation in H69AR cells (Supplementary Table S3). Consistent with this, TREX1 depletion did not affect the expression of *SLFN11* (Supplementary Fig. S4D), which is closely related to drug resistance in SCLC (36, 37). However, TREX1 depletion slightly lowered IC50 to DNA damaging agents (Supplementary Fig. S4E), and the combination of etoposide and TREX1 depletion synergistically decreased cell number (Supplementary Fig. S4F), which was partially rescued by overexpressing TREX1-WT, but not the TREX1-D18N mutant (Supplementary Fig. S4F). Furthermore, given that TREX1 depletion also triggered cGAS-STING pathway activation in drug-resistant cells (Fig. 3), we next evaluated p-IRF3 expression as a marker of innate immune response and observed that the expression of TREX1-WT, but not the D18N mutant, was able to rescue the induction of p-IRF3 observed after TREX1 depletion (Supplementary Fig. S4G), suggesting that DNA exonuclease activity of TREX1 is necessary to suppress innate immunity.

We next explored the impact of TREX1 upregulation on the growth of cisplatin-resistant SCLC cells *in vivo*. WT or TREX1-silenced H82-CispR cells were transplanted subcutaneously into the flanks of immunodeficient NSG mice and tumor growth rate was measured during systemic cisplatin treatment. sgTREX1 tumors showed a significant decrease in tumor volume and weight after treatment with cisplatin compared with Scr tumors (Fig. 4E; Supplementary Fig. S4H). Similar results were obtained by *in vivo* experiments comparing Scr and sgTREX1 H69AR cells (Fig. 4F; Supplementary Fig. S4I), suggesting that cytoplasmic dsDNA degradation by TREX1 contributes to the survival and growth of cisplatin-resistant SCLC cells. To further validate the effects of TREX1 depletion on cisplatin sensitivity *in vivo*, we next performed an experiment with an inducible shRNA knockdown system. We confirmed that *IFNB* gene expression was induced by DOX treatment in shTREX1 H1048 cells (Fig. 4G). Similar to the *in vivo* results using the CRISPR system, we observed that cisplatin treatment significantly decreased tumor growth in shTREX1 tumors when compared with shCtrl groups or untreated shTREX1 tumors (Fig. 4H), suggesting that cytoplasmic dsDNA degradation by TREX1 contributes to the survival and growth of chemoresistant SCLC cells. Together, these data provide additional support that TREX1 induction contributes to the survival capacity of SCLC cells as a strategy to suppress an innate immune activation triggered by dsDNAs derived from DNA-damaging agents (Fig. 4I), but additional mechanisms will play a role in SCLC chemoresistance, which will warrant further investigation.

### TREX1 expression is increased in SCLC patient samples postchemotherapy

**Figure 5:**
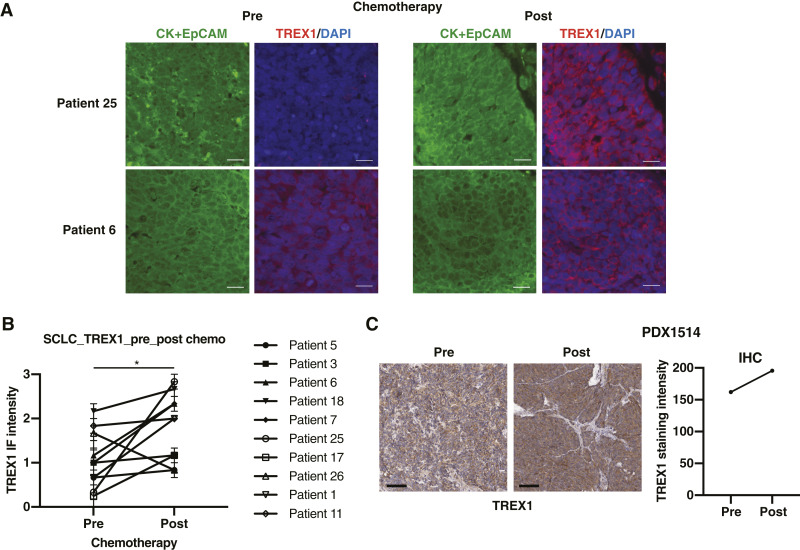
TREX1 expression is induced in posttreated human SCLC tumors. **A,** IF staining of CK + EpCAM (green), TREX1 (red) protein in patient 25 and patient 6 SCLC tumors are shown, pre- and post-chemotherapy treatment. **B,** TREX1 IF intensity in 10 patients is compared between pre- and post-chemotherapy treatment. **C,** TREX1 IHC intensity in a paired PDX tumor is compared between pre- and post-chemotherapy treatment. Scale bar, 100 μm. Data represent mean ± SEM. ns, not significant; *, *P* < 0.05; **, *P* < 0.01; ***, *P* < 0.001; ****, *P* < 0.0001 by paired Student *t* test (**B**).

To validate our findings in clinical settings, we next evaluated TREX1 protein levels by immunofluorescence (IF) across a panel of 10 paired human SCLC specimens collected before and after chemotherapy treatment (Table 1). TREX1 was mainly expressed on SCLC tumor cells which are positive for Cytokeratin and EpCAM (Fig. 5A). Remarkably, TREX1 levels were upregulated in 9 of 10 cases postchemotherapy (Fig. 5B).

**Table 1:** Information of patients with SCLC

Patient #	Age	Sex	Race	Stage	Chemotherapy regimen	Platinum response	Pre-chemo diagnostic	Post-chemo diagnostic
1	67	F	W	LS	Cisplatin/etoposide	S	Lung	Brain
3	84	F	W	LS	Cisplatin/etoposide	S	Lung	Brain
5	64	M	W	LS	Cisplatin/etoposide	S	Lung	Brain
6	51	M	W	LS	U	U	Lung	Brain
7	75	F	W	ES	Cisplatin/etoposide	S	Brain	Lung
11	58	F	W	LS	Cisplatin/etoposide	R	Lung	Brain
17	75	F	W	LS	Cisplatin/etoposide	S	Lung	Brain
18	76	F	W	ES	Cisplatin/etoposide	S	Lung	Brain
25	59	M	W	ES	Cisplatin/etoposide	R	Brain	Adrenal
26	64	F	W	ES	Cisplatin/etoposide	R	Brain	Adrenal

F, female; M, male; W, white; LS, limited stage; ES, extensive stage; S, sensitive; R, resistant; U, unknown.

Furthermore, to further validate the upregulation of TREX1 in SCLC tumors following chemotherapy, we additionally assessed TREX1 expression in matched PDX tumors derived from the lungs of the same patient with SCLC before and after treatment. Consistent with previous results in human SCLC specimens (Fig. 5B), TREX1 expression was higher in the PDX sample derived from a posttreatment tumor, at protein and mRNA levels (Fig. 5C; Supplementary Fig. S5A), which further suggests that TREX1 is induced after chemotherapy treatment in human SCLC.

## Discussion

A substantial proportion of SCLC tumors rapidly acquire therapeutic resistance despite their exceptional initial response (1). Here, we provide the first evidence that expression of TREX1 is highly induced in drug-resistant SCLC cells compared with untreated naive cancer cells. Notably, we identified TREX1 overexpression not only in chemoresistant SCLC cell lines but also in human SCLC tumors, confirmed by comparing TREX1 protein expression in pre- and post-chemotherapy paired-clinical samples. Mechanistically, we demonstrate that TREX1 upregulation mediates adaptive resistance to chemotherapy by suppressing cGAS-STING pathway activation due to the accumulation of dsDNA or other forms of chemotherapy-induced DNA damage. Targeting TREX1 in chemoresistant SCLC cells induces intrinsic innate immune activation, leading to compromised cancer cell viability. These findings position TREX1 as a novel and promising target to enhance antitumor immunity and overcome resistance to chemotherapy in SCLC.

TREX1 plays a crucial role in the innate immune response, and mutations in TREX1 are associated with autoimmune diseases such as Aicardi–Goutières syndrome, systemic lupus erythematosus, and familial chilblain lupus (30, 31, 38). This is because TREX1 malfunction results in the aberrant accumulation of cytoplasmic DNA, which triggers cGAS-STING pathway activation. However, little is known about the potential role of TREX1 in the regulation of antitumor immunity. Of note, a recent study demonstrated that TREX1 is induced in mouse carcinomas by high radiation doses to suppress type I IFN response, whereas it was not induced by low radiation treatment (15), indicating that TREX1 plays a role as an upstream regulator of radiation-driven antitumor immunity.

Our findings demonstrate that TREX1 depletion triggers an intrinsic innate immune response through cGAS-STING pathway activation in multiple SCLC chemoresistant models, which leads to decreased cell proliferation rate and survival. This could be attributed to the activation of the cGAS-STING pathway, which triggers the production of type I IFNs and other STAT1-driven effector programs that are cytotoxic. Of note, a previous report has revealed that DNA damage itself is not sufficient to induce an innate immune response. However, when cells with damaged DNA divide and micronuclei are formed, cGAS is recruited to the micronuclei, and STING is activated (33). Our data suggest that in chemoresistant SCLC cells, which are continuously exposed to chemotherapy-derived DNA damage, DNA exonucleases like TREX1 may play a crucial role as an adaptive resistance mechanism to suppress cGAS-STING pathway activation due to the constant accumulation of chemotherapy-induced micronuclei or other forms of cytosolic DNAs.

SCLC is often characterized by reduced antigen presentation and an immunosuppressive tumor microenvironment, resulting in failure to achieve a successful response to immunotherapy in most patients with SCLC (1, 29). Our data demonstrate that TREX1 depletion induces tumor cell-intrinsic antiviral signaling that translates into enhanced cell surface expression of MHC-I, as well as secretion of cytokines and chemokines that lead to T cell chemotaxis, suggesting that targeting TREX1 may be a promising strategy to maximize the immunogenicity of chemotherapy or chemoimmunotherapy in “cold” tumors, such as SCLC. However, we also observed an upregulation of some drug transporters, including ABCC3 and ABCC6, in H69AR chemoresistant cells (Supplementary Table S3). These ABC transporters are known to be involved in drug resistance as they can transport a broad range of endogenous and xenobiotic substrates including etoposide and cisplatin (39). Additionally, exogenous TREX1 expression was not sufficient to confer chemoresistance in drug-sensitive SCLC cells (Supplementary Fig. S4C). Although our findings suggest that TREX1 plays an important role in suppressing cGAS-STING pathway activation due to the accumulation of chemotherapy-induced DNA damage in drug-resistant SCLC, representing a promising target for inducing antitumor immune responses, a direct link between TREX1 upregulation and chemoresistance remains to be elucidated. Thus, future investigations are warranted to further clarify the role of TREX1 in chemoresistance.

Our study also reveals that chromatin accessibility of the TREX1 gene locus is highly increased in chemoresistant cells, as well as H3K27 acetylation, which results in TREX1 overexpression. However, how the TREX1 gene locus is epigenetically regulated and impacted by chemotherapy or other anticancer therapies remains unknown. Previous studies have shown that exposure to stress such as chemotherapy, immunotherapy, or targeted therapy in cancer cells can cause dramatic epigenetic alterations including DNA methylation, histone acetylation, and chromatin remodeling (40–42). Indeed, several studies have identified promising therapeutic targets, such as KDM5A (40), SETDB1/2 (43), or EZH2 (44), that could potentially overcome drug resistance caused by epigenetic mechanisms in SCLC or other tumors. However, the clinical significance of these targets remains unclear, and further research is needed to better understand when and how these epigenetic changes occur in SCLC treated with chemotherapy.

Our results support a cancer model whereby TREX1 upregulation is associated with chemotherapy resistance, as well as resistance to chemoimmunotherapy or ICB therapies by suppressing cGAS-STING-dependent innate immune response. Emerging data suggest that activating innate immunity in tumor cells by inducing intracellular accumulation of endogenous nucleic acids, including dsRNA and dsDNA, is an effective strategy to trigger an antitumor immune response and enhance cancer immunotherapy (45–48). Indeed, our data demonstrate that targeting TREX1 not only enhances the sensitivity of chemoresistant SCLC to chemotherapy *in vitro* and *in vivo* but also activates the cGAS-STING pathway, producing an antiviral response in cancer cells. Importantly, although multiple lines of investigation in academia and industry are pursuing the use of STING agonists as a therapeutic strategy to enhance tumor immunogenicity, it is tempting to speculate that direct inhibition of TREX1, acting upstream of STING, may produce an added benefit to amplify the signal producing a more robust interferon signal.

Although our findings elucidate a promising new target for chemoresistant SCLC, there are some limitations to this study. One major challenge is the limited accessibility to SCLC clinical tumor samples, especially after chemotherapy treatment or after clinical progression. Although we tested TREX1 expression across a panel of 10 paired human SCLC tumors collected before and after chemotherapy treatment, as well as matched PDX tumors, future studies with larger datasets will be necessary to confirm an association between TREX1 expression and chemoresistance. Additional matched PDX models representing chemosensitivity and chemoresistance in SCLC, as well as novel patient-derived culture platforms to study these tumors *ex vivo* will be promising strategies to better understand SCLC chemoresistance.

Furthermore, although we demonstrated that TREX1 contributed to the survival and growth of cisplatin-resistant SCLC cells in xenograft models, the association of TREX1 and chemoresistance is still limited because of the lack of a chemoresistant SCLC model in an immunocompetent system, in which the impact of an immune response triggered by TREX1 depletion might be relevant. Thus, future efforts to develop syngeneic models of chemoresistant SCLC will be extremely beneficial. These models will not only enable further investigation into the role of TREX1 in chemoresistant SCLC within an intact immune system but will also help elucidate the broader mechanisms underlying chemoresistance in this complex disease.

Overall, this study reveals a novel mechanism by which chemoresistant SCLC cells suppress the cGAS-STING pathway to adapt and survive to the aberrant accumulation of chemotherapy-derived cytosolic DNA. Thus, consistent with recent studies highlighting the role of TREX1 on antitumor immunity in different cancer contexts (20, 49, 50), targeting TREX1 represents a promising strategy to turn immunologically “cold” tumors, such as chemoresistant SCLC, into “hot” tumors, potentially enhancing the response to chemoimmunotherapy or ICB therapies and leading to long-term patient responses in this treatment-refractory tumor type.

## Supplementary Material

Figure S1 Figure S1 shows TREX1 expression is induced in drug resistant SCLC cells

Figure S2 Figure S2 shows TREX1 loss suppresses SCLC growth and induces immune response

Figure S3 Figure S3 shows immunogenicity is induced in TREX1 depleted cells

Figure S4 Figure S4 shows TREX1 depletion increases sensitivity of resistant SCLC cells to chemotherapy

Figure S5 Figure S5 shows TREX1 expression is induced in post-treated SCLC PDX

Table S1 qPCR primer sequences

Table S2 Treatment history of PDX tumors

Table S3 Drug transporter expression levels in H69AR *vs* H69
