# Supplementary material for: Targeting TREX1 Induces Innate Immune Response in Drug-Resistant Small-Cell Lung Cancer
Source: Cancer Res Commun. 2024 Sep 12;4(9):2399–414. doi: 10.1158/2767-9764.CRC-24-0360 (PMC11391691; doi:10.1158/2767-9764.CRC-24-0360)
Supplement: Figure S1 — shows TREX1 expression is induced in drug resistant SCLC cells [file crc-24-0360_figure_s1_suppsf1.pdf]

Sup Figure 1

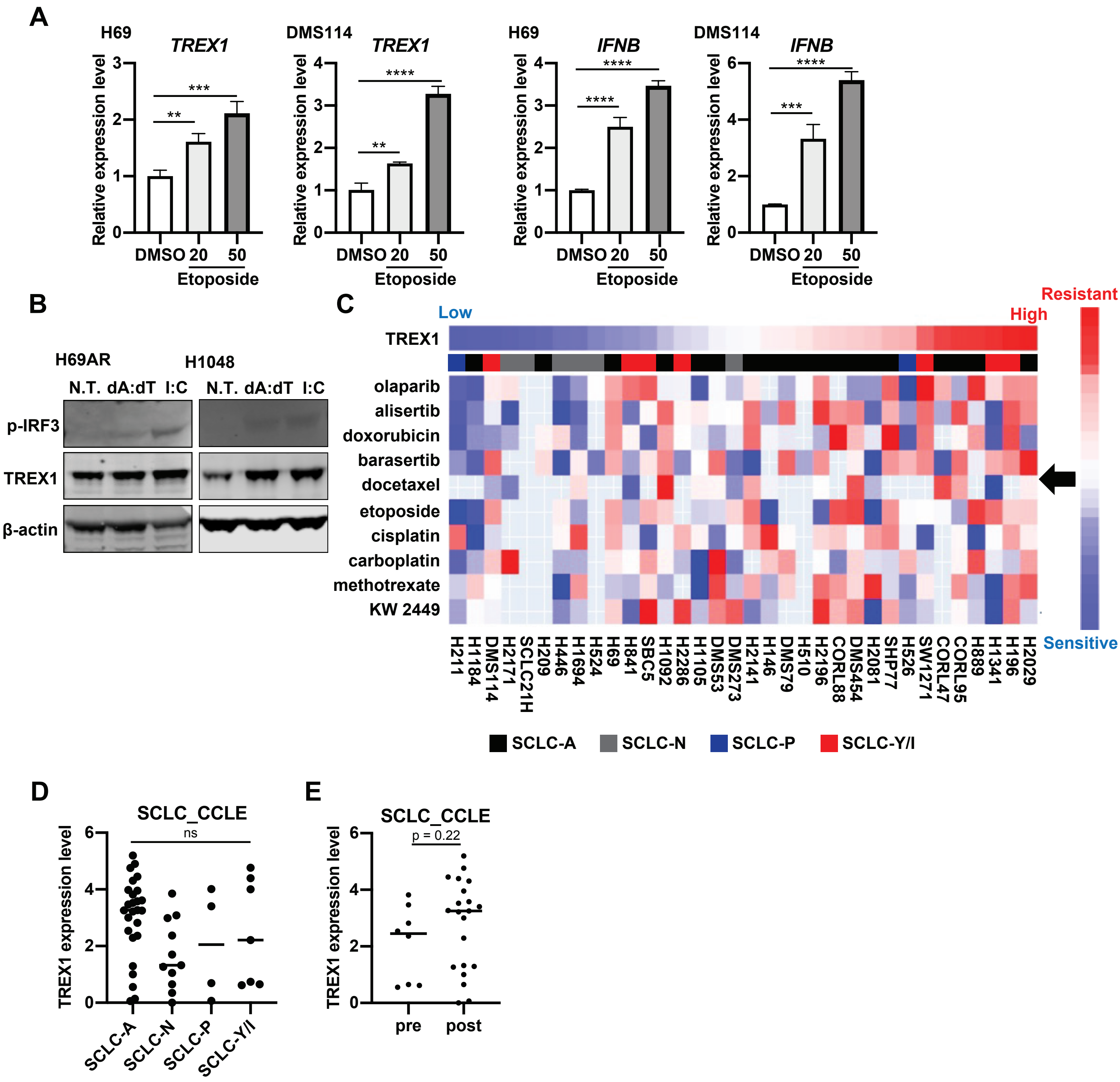
A
H69
TREX1
DMS114
TREX1
H69
IFNB
DMS114
IFNB
Relative expression level
DMSO 20 50
Etoposide
B
H69AR
H1048
N.T. dA:dT I:C
N.T. dA:dT I:C
p-IRF3
TREX1
β-actin
C
Low
High
TREX1
Resistant
Sensitive
olaparib
alisertib
doxorubicin
barasertib
docetaxel
etoposide
cisplatin
carboplatin
methotrexate
KW 2449
H211
H1184
DMS114
H2171
SCLC21H
H209
H446
H1694
H524
H69
H841
SBC5
H1092
H2286
H1105
DMS53
DMS273
H2141
H146
DMS79
H510
H2196
CORL88
DMS454
H2081
SHP77
H526
SW1271
CORL47
CORL95
H889
H1341
H196
H2029
SCLC-A
SCLC-N
SCLC-P
SCLC-Y/I
D
SCLC_CCLE
TREX1 expression level
ns
SCLC-A
SCLC-N
SCLC-P
SCLC-Y/I
E
SCLC_CCLE
TREX1 expression level
p = 0.22
pre
post

Figure Legends

Supplementary Figure S1.

A, Expression levels of *TREX1* and *IFNB* genes in H69, DMS114 cells treated with Etoposide (20 or 50 μM) or DMSO were compared by qPCR (mean ± SEM, n = 3). **B**, Expression levels of TREX1 and p-IRF3 in H69AR, H1048 cells treated w/wo poly(dA:dT) or poly(I:C) were compared by immunoblotting. **C**, Drug sensitivity/resistance data of SCLC cell lines are summarized. Cells are lined up based on *TREX1* expression levels. **D**, Expression levels of *TREX1* gene were compared among SCLC-A, -N, -P and -Y/I subtypes. **E**, Expression levels of *TREX1* gene were compared between pre- and post- treated tumor-derived cell lines.

Data represent mean ± SEM. ns, not significant; *p < 0.05, **p < 0.01, ***p < 0.001, ****p < 0.0001 by one-way ANOVA followed by Dunnett's multiple comparisons test (A and D), and Mann-Whitney test (E).
