# Supplementary material for: Targeting TREX1 Induces Innate Immune Response in Drug-Resistant Small-Cell Lung Cancer
Source: Cancer Res Commun. 2024 Sep 12;4(9):2399–414. doi: 10.1158/2767-9764.CRC-24-0360 (PMC11391691; doi:10.1158/2767-9764.CRC-24-0360)
Supplement: Figure S2 — shows TREX1 loss suppresses SCLC growth and induces immune response [file crc-24-0360_figure_s2_suppsf2.pdf]

Sup Figure 2

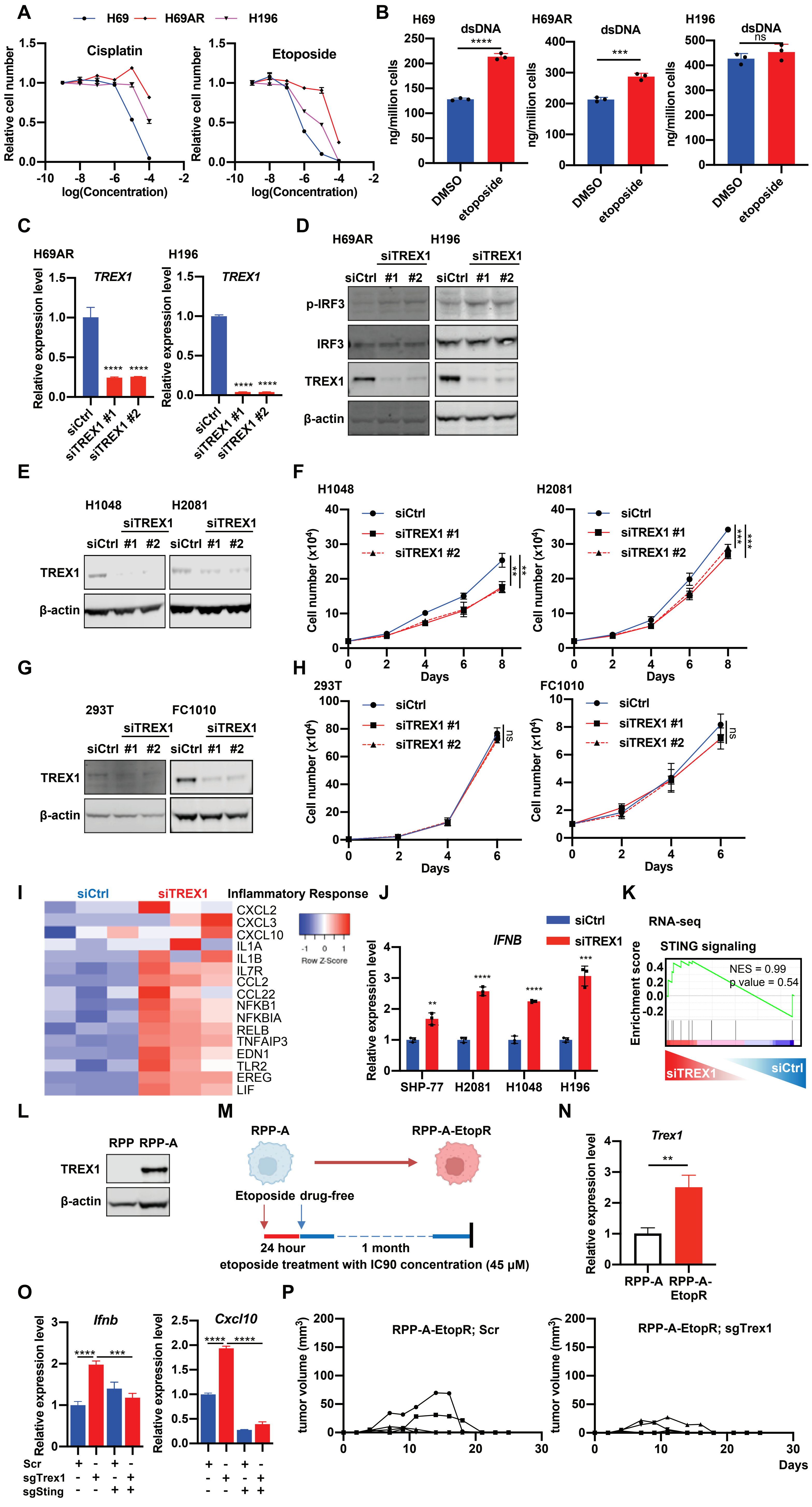
A
H69
H69AR
H196
Cisplatin
Etoposide
Relative cell number
log(Concentration)
B
H69
H69AR
H196
dsDNA
ng/million cells
****
***
ns
DMSO
etoposide
C
H69AR
H196
TREX1
Relative expression level
siCtrl
siTREX1 #1
siTREX1 #2
D
H69AR
H196
siTREX1
siCtrl
#1
#2
p-IRF3
IRF3
TREX1
β-actin
E
H1048
H2081
F
H1048
H2081
siCtrl
siTREX1 #1
siTREX1 #2
Cell number (x10⁴)
Days
G
293T
FC1010
H
293T
FC1010
I
siCtrl
siTREX1
Inflammatory Response
CXCL2
CXCL3
CXCL10
IL1A
IL1B
IL7R
CCL2
CCL22
NFKB1
NFKBIA
RELB
TNFAIP3
EDN1
TLR2
EREG
LIF
Row Z-Score
J
IFNB
siCtrl
siTREX1
SHP-77
H2081
H1048
H196
K
RNA-seq
STING signaling
NES = 0.99
p value = 0.54
Enrichment score
siTREX1
siCtrl
L
RPP
RPP-A
TREX1
β-actin
M
RPP-A
RPP-A-EtopR
Etoposide drug-free
24 hour
1 month
etoposide treatment with IC90 concentration (45 μM)
N
Trex1
RPP-A
RPP-A-EtopR
O
Ifnb
Cxcl10
Scr
sgTrex1
sgSting
P
RPP-A-EtopR; Scr
RPP-A-EtopR; sgTrex1
tumor volume (mm³)
Days

Supplementary Figure S2.

A, Viability of H69, H69AR and H196 cells after 5 days of Cisplatin or Etoposide exposure was assessed using the CellTiter-Glo Cell Viability Assay. **B**, Cytoplasmic dsDNA amounts in H69, H69AR and H196 cells were compared between DMSO and Etoposide treated conditions, using SpectraMax Quant dsDNA Assay Kit (mean ± SEM, n = 3). **C**, Expression levels of *TREX1* gene in H69AR and H196 were compared between siCtrl and siTREX1 (#1 and #2) transfected cells by qPCR (mean ± SEM, n = 3). **D**, Expression levels of TREX1, IRF3, and p-IRF3 in H69AR and H196 were compared by immunoblotting between cells transfected with siCtrl and siTREX1 (#1 and #2). **E**, Expression levels of TREX1 in H1048 and H2081 cells were compared by immunoblotting between cells transfected with siCtrl and siTREX1 (#1 and #2). **F**, Growth curves of H1048 and H2081 cells were compared between siCtrl and siTREX1 (#1 and #2) (mean ± SEM, n = 3). **G**, Expression levels of TREX1 in HEK293T and FC1010 cells were compared by immunoblotting between cells transfected with siCtrl and siTREX1 (#1 and #2). **H**, Growth curves of HEK293T and FC1010 cells were compared between siCtrl and siTREX1 (#1 and #2) (mean ± SEM, n = 3). **I**, Heat map of RNA-seq results comparing siCtrl and siTREX1 H69AR cells. **J**, Expression levels of *IFNB* gene in SHP-77, H2081, H1048 and H196 cells were compared between siCtrl and siTREX1 transfected cells by qPCR (mean ± SEM, n = 3). **K**, GSEA analysis with C2-registered STING hallmark gene set, based on RNA-seq results of siTREX1 versus siCtrl H69AR cells. **L**, Expression levels of TREX1 in RPP and RPP-A cells were compared by immunoblotting. **M**, Schematic of method used to establish Etoposide-resistant RPP-A (RPP-A-EtopR) cells. **N**, Expression levels of *Trex1* gene in RPP-A and RPP-A-EtopR cells were compared by qPCR (mean ± SEM, n = 3). **O**, Expression levels of *Ifnb* and *Cxcl10* genes in Scr and sgTrex1 cells w/wo Sting knockout were compared by qPCR (mean ± SEM, n = 3). **P**, Tumor growth curves of Scr and sgTrex1 RPP-A-EtopR tumors (n = 4) were compared.

Data represent mean ± SEM. ns, not significant; $*p < 0.05$, $**p < 0.01$, $***p < 0.001$, $****p < 0.0001$ by unpaired Student's t test (B, J and N), one-way ANOVA followed by Dunnett's multiple comparisons test (C, F and H), and two-way ANOVA followed by Tukey's multiple comparisons test (O).
