# Supplementary material for: Targeting TREX1 Induces Innate Immune Response in Drug-Resistant Small-Cell Lung Cancer
Source: Cancer Res Commun. 2024 Sep 12;4(9):2399–414. doi: 10.1158/2767-9764.CRC-24-0360 (PMC11391691; doi:10.1158/2767-9764.CRC-24-0360)
Supplement: Figure S3 — shows immunogenicity is induced in TREX1 depleted cells [file crc-24-0360_figure_s3_suppsf3.pdf]

Sup Figure 3

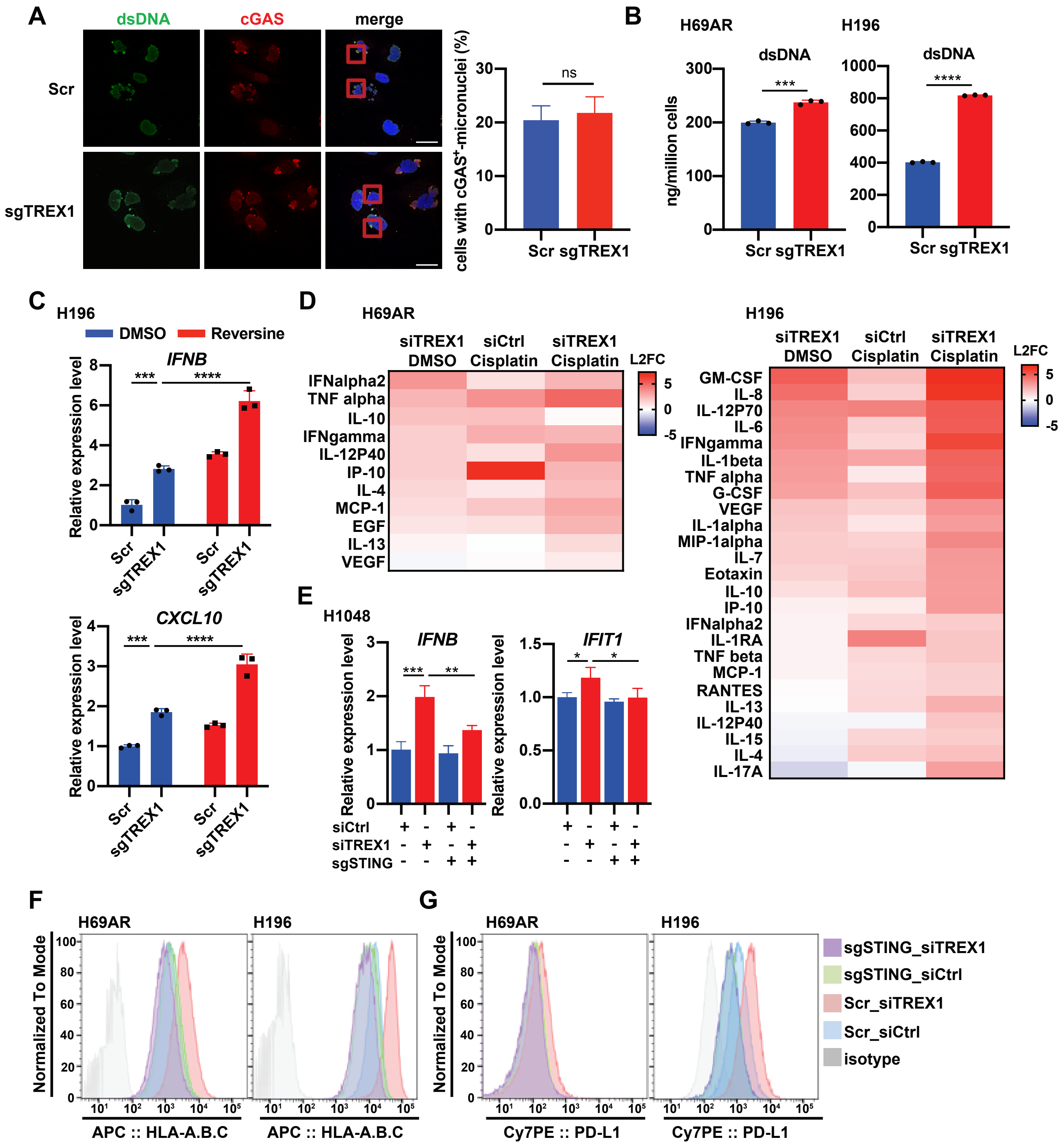

A
dsDNA
cGAS
merge
Scr
sgTREX1
cells with cGAS+-micronuclei (%)
ns
Scr sgTREX1
B
H69AR
H196
dsDNA
ng/million cells
***
****
Scr sgTREX1
C
H196
DMSO
Reversine
IFNB
CXCL10
Relative expression level
***
****
Scr
sgTREX1
D
H69AR
siTREX1 DMSO
siCtrl Cisplatin
siTREX1 Cisplatin
L2FC
IFNalpha2
TNF alpha
IL-10
IFNgamma
IL-12P40
IP-10
IL-4
MCP-1
EGF
IL-13
VEGF
H196
GM-CSF
IL-8
IL-12P70
IL-6
IFNgamma
IL-1beta
TNF alpha
G-CSF
VEGF
IL-1alpha
MIP-1alpha
IL-7
Eotaxin
IL-10
IP-10
IFNalpha2
IL-1RA
TNF beta
MCP-1
RANTES
IL-13
IL-12P40
IL-15
IL-4
IL-17A
E
H1048
IFNB
IFIT1
Relative expression level
siCtrl
siTREX1
sgSTING
F
G
H69AR
H196
Normalized To Mode
APC :: HLA-A.B.C
Cy7PE :: PD-L1
sgSTING_siTREX1
sgSTING_siCtrl
Scr_siTREX1
Scr_siCtrl
isotype

Supplementary Figure S3.

A, (Left) Immunofluorescence images of cGAS (red) and dsDNA (green) staining of Scr and sgTREX1 H196 cells are shown. Nuclei were counterstained with DAPI. Scale bar = 50 μm. (Right) Proportion of cells with $cGAS^{+}$-micronuclei was compared (> 150 cells were counted, n = 3). **B**, Cytoplasmic dsDNA amounts in H69AR and H196 were compared between Scr and sgTREX1 conditions, using SpectraMax Quant dsDNA Assay Kit (mean ± SEM, n = 3). **C,** Expression levels of *IFNB* and *CXCL10* genes in Scr or sgTREX1 H196 cells, treated with 0.5 μM Reversine or DMSO for 72 hours were compared by qPCR (mean ± SEM, n = 3). **D**, Log2 fold change (L2FC) cytokine/chemokine differences of siTREX1-DMSO, siCtrl-Cisplatin and siTREX1-Cisplatin with the control (siCtrl-DMSO) were shown. The cytokine/chemokine levels were quantified with Proteome Profiler Human Cytokine Array Kit. **E**, Expression levels of *IFNB* and *IFIT1* genes were compared between H1048 cells transduced with Scr or STING sgRNA, after transfection with siCtrl or siTREX1 (#1) (mean ± SEM, n = 3). **F and G**, HLA-A, B, C (F) and PD-L1 (G) expressions in H69AR and H196 cells transduced with STING or Scr sgRNA, after transfection with siCtrl or siTREX1 (#1), were tested by flow cytometry.

Data represent mean ± SEM. ns, not significant; *p < 0.05, **p < 0.01, ***p < 0.001, ****p < 0.0001 by unpaired Student's t test (A and B), and two-way ANOVA followed by Tukey's multiple comparisons test (C and E).
