# Supplementary material for: Targeting TREX1 Induces Innate Immune Response in Drug-Resistant Small-Cell Lung Cancer
Source: Cancer Res Commun. 2024 Sep 12;4(9):2399–414. doi: 10.1158/2767-9764.CRC-24-0360 (PMC11391691; doi:10.1158/2767-9764.CRC-24-0360)
Supplement: Figure S4 — shows TREX1 depletion increases sensitivity of resistant SCLC cells to chemotherapy [file crc-24-0360_figure_s4_suppsf4.pdf]

# Sup Figure 4

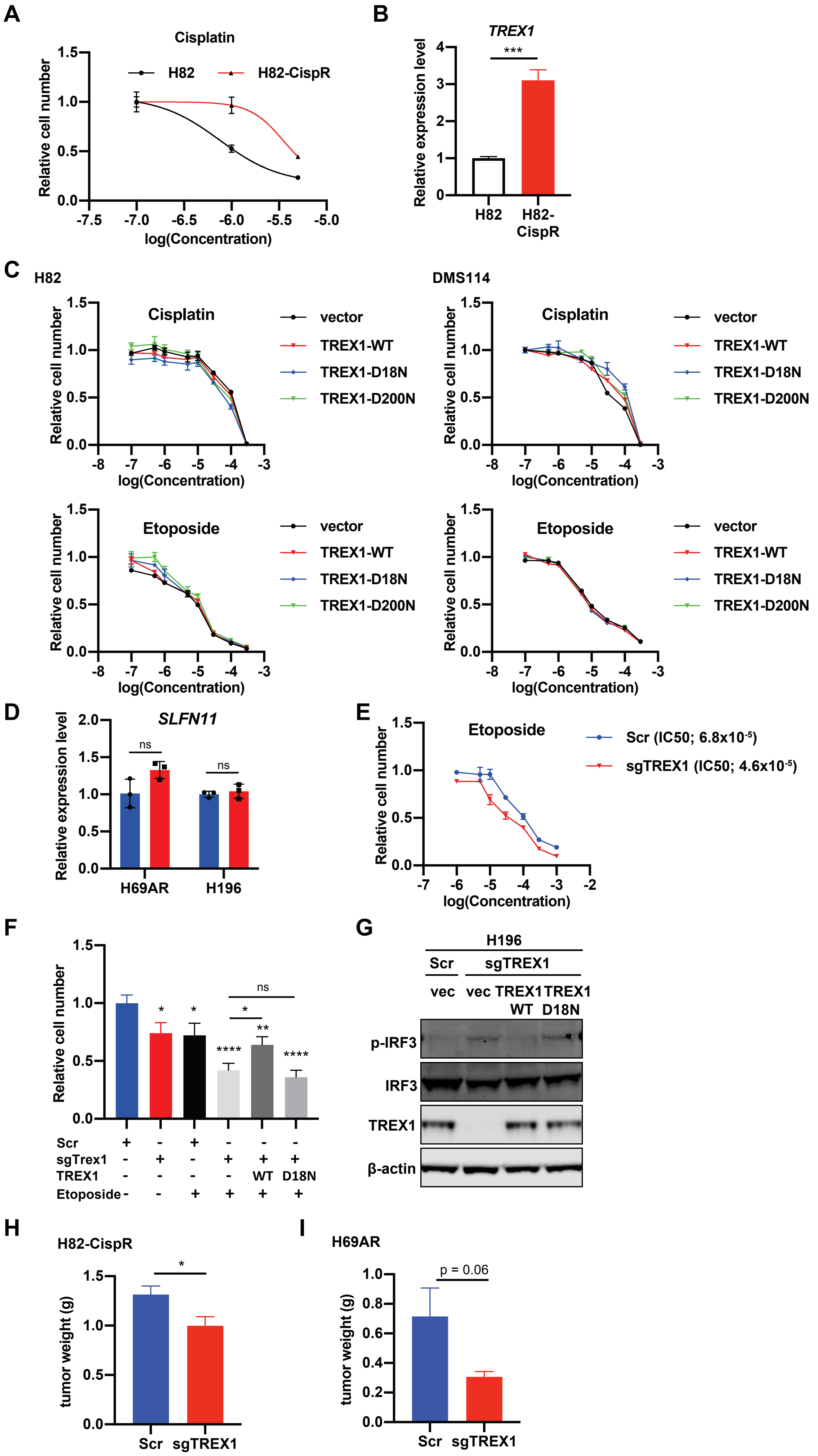
A
Cisplatin
H82
H82-CispR
Relative cell number
1.5
1.0
0.5
0.0
-7.5
-7.0
-6.5
-6.0
-5.5
-5.0
log(Concentration)
B
TREX1
***
Relative expression level
4
3
2
1
0
H82
H82-CispR
C
H82
DMS114
Cisplatin
Etoposide
vector
TREX1-WT
TREX1-D18N
TREX1-D200N
Relative cell number
-8
-7
-6
-5
-4
-3
log(Concentration)
D
SLFN11
Relative expression level
2.0
1.5
1.0
0.5
0.0
ns
ns
H69AR
H196
E
Etoposide
Scr (IC50; 6.8x10-5)
sgTREX1 (IC50; 4.6x10-5)
Relative cell number
-7
-6
-5
-4
-3
-2
log(Concentration)
F
Relative cell number
ns
*
**
****
****
Scr
sgTrex1
TREX1
Etoposide
WT
D18N
G
H196
Scr
sgTREX1
vec
vec
TREX1 WT
TREX1 D18N
p-IRF3
IRF3
TREX1
β-actin
H
H82-CispR
*
tumor weight (g)
1.5
1.0
0.5
0.0
Scr
sgTREX1
I
H69AR
p = 0.06
tumor weight (g)
1.0
0.8
0.6
0.4
0.2
0.0
Scr
sgTREX1

**Supplementary Figure S4.**

**A**, Viability of H82 and H82-CispR cells after 5 days of Cisplatin exposure was assessed using the CellTiter-Glo Cell Viability Assay. **B**, Expression levels of *TREX1* gene in H82 and H82-CispR cells were compared by qPCR (mean ± SEM, n = 3). **C**, Viability of H82, DMS114 (vector, TREX1-WT/D18N/D200N) cells after 2 days of Cisplatin/Etoposide exposure was assessed using the CellTiter-Glo Cell Viability Assay. **D**, Expression levels of *SLFN11* gene were compared between siCtrl and siTREX1 (#1) transfected cells by qPCR (mean ± SEM, n = 3). **E**, Viability of H196 (Scr, sgTREX1) cells after 2 days of Etoposide exposure was assessed using the CellTiter-Glo Cell Viability Assay. **F**, Relative cell number of H196 (Scr, sgTREX1, sgTREX1+TREX1-WT, sgTREX1+TREX1-D18N) w/wo 4 days treatment of Etoposide (10 μM). **G**, Expression levels of TREX1, IRF3, and p-IRF3 in H196 were compared by immunoblotting between cells transduced with Scr and sgTREX1, w/wo TREX1-WT/D18N. **H**, Tumor weights of Scr and sgTREX1 H82-CispR tumors (n = 5; collected on Day 23), were compared. **I**, Tumor weights of Scr and sgTREX1 H69AR tumors (n = 6; collected on Day 23), were compared.

Data represent mean ± SEM. ns, not significant; *p < 0.05, **p < 0.01, ***p < 0.001, ****p < 0.0001 by unpaired Student's t test (B, D, H and I), and one-way ANOVA followed by Dunnett's multiple comparisons test (F).
