# Supplementary material for: Targeting TREX1 Induces Innate Immune Response in Drug-Resistant Small-Cell Lung Cancer
Source: Cancer Res Commun. 2024 Sep 12;4(9):2399–414. doi: 10.1158/2767-9764.CRC-24-0360 (PMC11391691; doi:10.1158/2767-9764.CRC-24-0360)
Supplement: Figure S5 — shows TREX1 expression is induced in post-treated SCLC PDX [file crc-24-0360_figure_s5_suppsf5.pdf]

Sup Figure 5

A

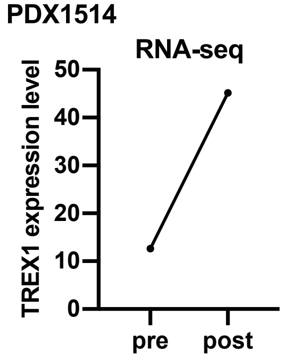

Supplementary Figure S5.

A, TREX1 expression levels (RNA-seq) in PDX tumors are compared between pre- and post-chemotherapy treatment samples. Expression data were downloaded from the GEO database (GSE110853).
